# USP7 promotes endothelial activation to aggravate sepsis-induced acute lung injury through PDK1/AKT/NF-κB signaling pathway

**DOI:** 10.1038/s41420-025-02481-1

**Published:** 2025-04-17

**Authors:** Zhiyi Liu, Xiaoyun Shi, Tiantian Ke, Zhisu Yan, Lei Xiong, Fang Tang

**Affiliations:** Department of Anesthesiology and Operative Medicine, Medical Center of Anesthesiology and Pain, The First Affiliated Hospital, Jiangxi Medical College, Nanchang University, Nanchang, Jiangxi China

**Keywords:** Acute inflammation, Ubiquitylation

## Abstract

Disruption of the endothelial cell barrier and the subsequent inflammatory response represent a central pathological feature of acute lung injury (ALI). Ubiquitination plays a pivotal role in regulating protein stability, intracellular transport, and enzyme activity, which is typically reversed by deubiquitinating enzymes. Nevertheless, the function of deubiquitinating enzymes in endothelial biology and in ALI remains largely uninvestigated. The present study demonstrates that the expression of USP7 is increased in instances of endothelial inflammation and ALI. The knockdown or inhibition of USP7 using specific inhibitors was observed to significantly reduce the TNF-α-induced inflammatory response of endothelial cells and their adhesion capacity to monocytes. Conversely, the overexpression of USP7 was observed to promote the inflammatory response and adhesion capacity of endothelial cells. The specific inhibitors of USP7 were found to be effective in mitigating acute lung injury induced by LPS. From a mechanistic perspective, our findings indicate that USP7 binds and deubiquitinates PDK1, thereby stabilizing PDK1 and promoting the activity of the inflammatory pathway in endothelial cells. In conclusion, our findings demonstrate the role of a novel USP7-PDK1 signaling axis in regulating TNF-α-induced vascular endothelial injury and reveal that USP7 is a deubiquitylating enzyme of PDK1. These observations suggest that targeting the USP7-PDK1 axis may offer a promising therapeutic strategy for the treatment of acute lung injury.

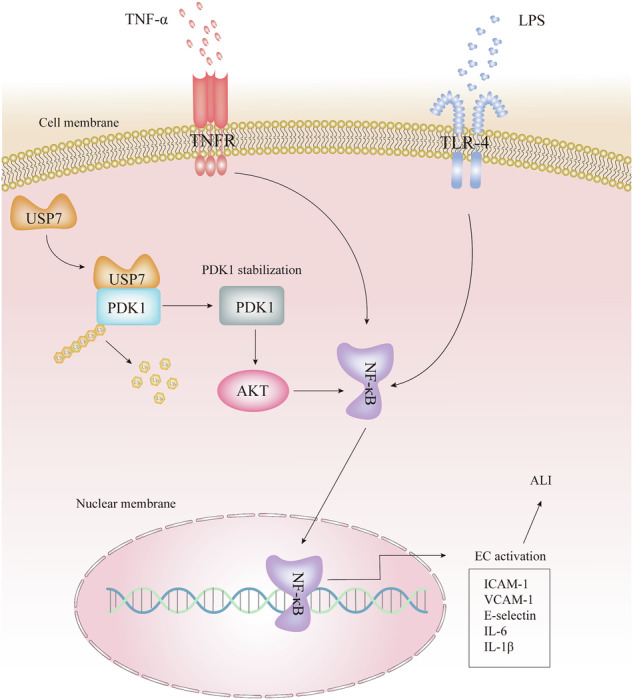

## Introduction

The lung is a vital immune organ in mammals, serving as a primary target for a multitude of pathogens, toxins, and allergens [1]. Sepsis can result in damage to lung epithelial and endothelial cells, leading to the release of pro-inflammatory molecules and increased endothelial permeability, which ultimately contributes to the development of acute lung injury (ALI) [2]. ALI is a life-threatening hypoxic respiratory disease, the most severe forms of which include acute respiratory distress syndrome (ARDS), with a mortality rate of up to 40% [3]. At present, the treatment of ALI comprises non-pharmacological interventions, such as lung-protective mechanical ventilation and prone positioning, and pharmacological treatments, including neuromuscular blocking agents and anti-inflammatory drugs [4]. However, the mortality rate of ALI remains high.

Human pulmonary microvascular endothelial cells (HPMECs) constitute a selective vascular barrier and are a primary target during lung injury [5]. In normal physiological conditions, the pulmonary endothelium exerts an inhibitory effect on the inflammatory process. Nevertheless, the activation of the pulmonary endothelium in response to a multitude of pathological injuries, including hypoxia, bacterial toxins, and stimulation by pro-inflammatory cytokines and chemokines, results in the recruitment of inflammatory cells to the vessel wall and migration of leukocytes into the lung [5]. It is therefore evident that therapeutic strategies aimed at reducing endothelial activation are of great importance in order to reduce the incidence and severity of ARDS and other inflammatory vascular diseases [6]. The search for biomarkers of abnormal endothelial activation can therefore help in the accurate diagnosis and targeted treatment of ALI.

Numerous studies have shown that the ubiquitin-proteasome system (UPS) maintains protein homeostasis by controlling a variety of enzymes and is involved in a variety of cellular functions such as cell cycle, gene transcription, apoptosis and DNA repair [7]. Ubiquitination is a key post-translational modification that binds ubiquitin molecules to target protein substrates via E3 ubiquitin ligases, which are then recognized and degraded by the proteasome [8]. Deubiquitination is its reverse process, and these two processes maintain a dynamic balance that regulates important biological processes in the human body [8]. To date, about 100 deubiquitinating enzymes (DUBs) have been identified that remove ubiquitin from substrates, thereby terminating ubiquitin-dependent signaling pathways [9].

Ubiquitin-specific protease 7 (USP7), also known as herpes virus-associated ubiquitin-specific protease (HAUSP), is a member of the largest subfamily of deubiquitinating enzymes, the ubiquitin-specific protease (USP) family, which is involved in a variety of diseases by regulating the cell cycle, DNA repair, chromatin remodeling, and epigenetic regulation [10]. It has been shown that USP7 mediates pathophysiological angiogenesis by regulating the Sp1/Sp3-Notch1 signaling pathway in response to angiotensin-converting enzyme inhibitor (ACEI) [11]. Recently, our research has revealed that USP7 plays a role in the regulation of pulmonary fibrosis by targeting the TGF-β signaling pathway [12]. Of particular note is the fact that the TGF-β signaling pathway has been shown to promote sepsis-induced ferroptosis of lung epithelial cells in ALI through transcriptional regulation of USP7 [13]. However, the role and mechanism of USP7 in endothelial cell activation and ALI are unclear.

In this study, we reveal that USP7 expression is upregulated in endothelial cell activation and ALI. Knockdown of USP7 expression in endothelial cells inhibited endothelial cell inflammatory response and adhesion to monocytes and vice versa. USP7 inhibitors (P22077 and GNE-6776) can suppressed the activation of endothelial cell as well. Mechanistically, USP7 binds and deubiquitinates PDK1, stabilizes its protein level, and in turn promotes the AKT/NF-κB signaling pathway. The USP7 inhibitor P22077 effectively inhibited LPS-induced ALI. Thus, USP7 plays an important role in ALI by regulating endothelial cell activation, and targeting USP7 is a potential strategy for effective treatment of ALI.

## Results

### USP7 expression is elevated by inflammatory stimulation in endothelial cells

**Fig. 1:**
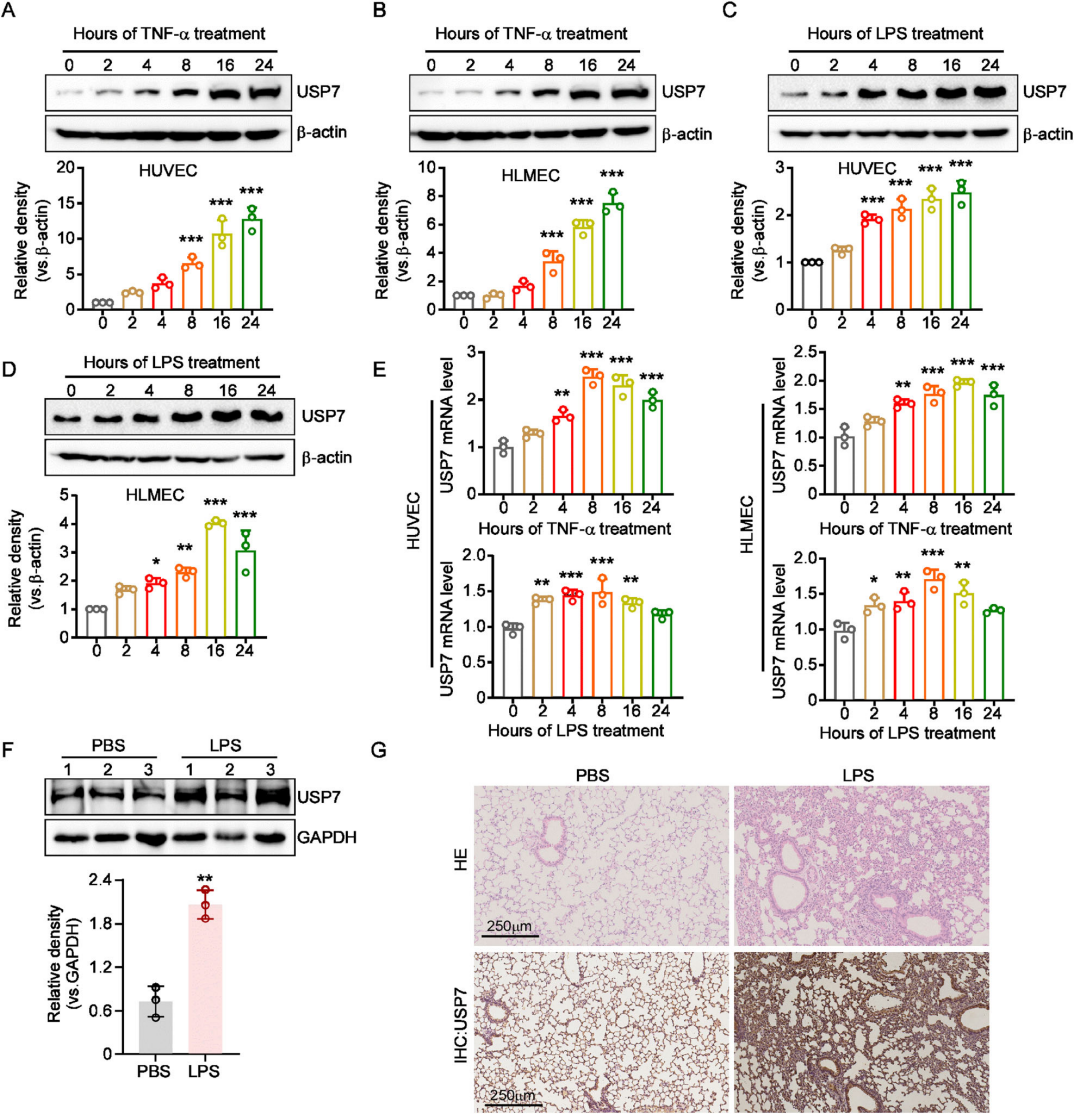
USP7 was induced in EC activation. **A** The expression of USP7 in HUVECs treated with 10 ng/mL TNFα at the indicated times was assessed by western blot, with β-actin serving as the loading control; **B** The expression of USP7 in HLMECs treated with 10 ng/mL TNFα was assessed by western blot; **C** Western blot analysis was performed on UVECs treated with 100 ng/mL LPS at the indicated times; **D** Western blot analysis was also performed on HLMECs treated with 100 ng/mL LPS at the indicated times; **E** The mRNA levels of USP7 in HUVECs or HLMECs after treatment of TNFα/LPS were detected by RT-qPCR, with β-actin as the reference gene; **F** Expression of USP7 in lungs of mice in control or LPS-treated groups was detected by western blot, with GAPDH serving as the loading control. **G** Representative images of HE staining and USP7 IHC analysis in mouse lung tissues from control and ALI groups. The relative protein density was determined using Gel-Pro Analyzer software, with β-actin used as the reference protein. All data were expressed as the mean ± standard deviation. Statistical significance was indicated by *P < 0.05, **P < 0.01, and ***P < 0.001.

To investigate the role of USP7 in the progression of ALI, we initially sought to detect the expression of USP7 in the activation of endothelial cells (ECs). As shown in Fig. 1A, B, the protein levels of USP7 can be induced by TNFα in a time-dependent manner in both HUVECs and HLMECs. Furthermore, the protein expression of USP7 was found to be elevated following LPS treatment in ECs (Fig. 1C, D). The results of the qPCR analysis demonstrated that the mRNA levels of USP7 were increased upon the addition of TNFα and LPS (Fig. 1E). Subsequently, the protein levels of USP7 were evaluated in lung tissues using western blot and IHC after LPS challenge to examine whether its expression was induced in vivo. As illustrated in Fig. 1F, G, the protein levels of USP7 were significantly elevated in lungs, which was consistent with the results observed in ECs. Collectively, these findings indicate that USP7 is highly expressed in ECs and can be induced by inflammatory stimuli both in vitro and in vivo, suggesting that it may play a role in regulating vascular endothelial activation.

### USP7 aggravate TNFα-induced endothelial activation

**Fig. 2:**
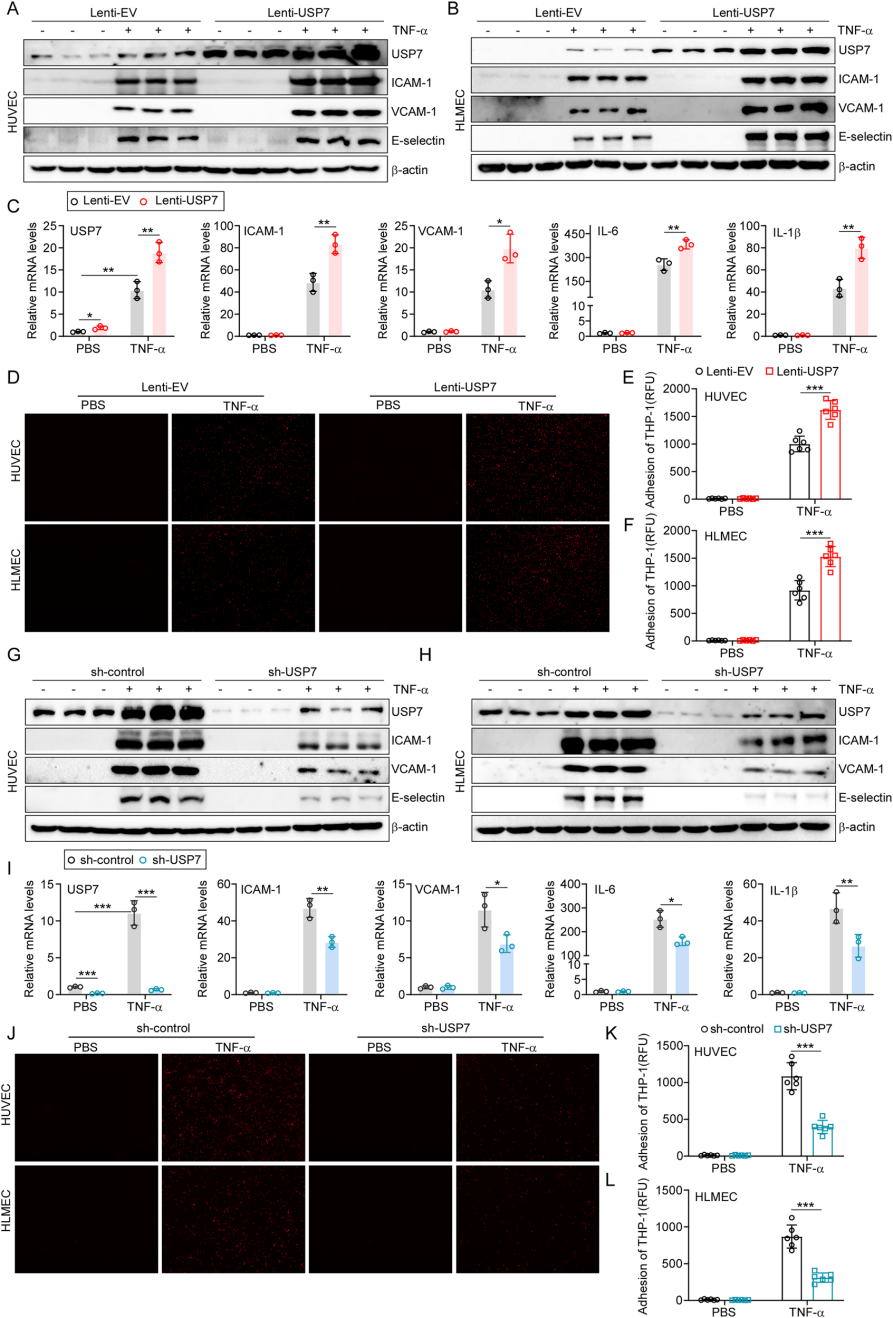
USP7 promoted the activation of ECs. **A** USP7 stably overexpressing HUVECs and control cells were treated with 10 ng/mL TNFα for 8 h, and then cell lysates were collected to detect the protein levels of adhesive molecules, including ICAM-1, VCAM-1 and E-selectin; **B** HLMECs stably overexpressing USP7 and control cells were treated with 10 ng/mL TNFα for 8 h, and then cell lysates were collected to detect the protein levels of those adhesive molecules as indicated; **C** After 4 h of TNFα treatment, the mRNA levels of adhesive molecules and cytokines were determined by qPCR in HUVECs; **D** Upon incubation with TNFα for 8 h, indicated HUVECs and HLMECs were co-cultured with PKH-26 labeled THP-1 cells for 1 h and observed under fluorescent microscope; **E** Attached cells in HUVECs were counted from six random pictures; **F** Attached cells in HLMECs were counted from six random pictures; **G** USP7 stably knockdown HUVECs and control cells were treated with 10 ng/mL TNFα for 8 h, and then cell lysates were collected to detect the protein levels of adhesive molecules, including ICAM-1, VCAM-1 and E-selectin; **H** USP7 stably knocking-down HLMECs and control cells were treated with 10 ng/mL TNFα for 8 h, and then cell lysates were collected to detect the protein levels of those adhesive molecules as indicated; **I** After 4 h of TNFα treatment, the mRNA levels of adhesive molecules and cytokines were determined by qPCR in HUVECs; **J** Upon incubation with TNFα for 8 h, indicated HUVECs and HLMECs were co-cultured with PKH-26 labeled THP-1 cells for 1 h and observed under fluorescent microscope; **K** Attached cells in HUVECs were counted from six random pictures; **L** Attached cells in HLMECs were counted from six random pictures. All data were expressed as mean ± SD. *P < 0.05, **P < 0.01, ***P < 0.001.

To investigate the function of USP7 in EC activation, we generated stable overexpressing or knockdown ECs via lenti-virus infection, respectively. The HUVECs and HLMECs were treated with 10 ng/mL TNFα, and the expression of adhesion molecules and cytokines was examined by western blot and qPCR. Overexpression of USP7 was observed to markedly increase the protein levels of some key adhesive molecules, including ICAM-1, VCAM-1 and E-selectin both in HUVECs and HLMECs (Figs. 2A, B and S1A, B). Following a four-hour incubation period with TNFα, significant facilitation of ICAM-1, VCAM-1, cytokine IL-6 and IL-1β transcription was observed (Fig. 2C). Given that activated endothelial cells are capable of recruiting monocytes from the bloodstream to their surface, we proceeded to investigate the impact of USP7 on monocyte adhesion to ECs. The activated and control ECs were incubated with fluorescence-labelled THP-1 cells, and after washing, the adherent THP-1 cells were imaged and counted. As illustrated in Fig. 2D–F, overexpression of USP7 was observed to enhance THP-1 adherence to HUVECs and HLMECs.

To further confirm the role of USP7 in the activation of ECs, loss-of-function studies were conducted using RNA interference (RNAi). As illustrated in Fig. 2G, H, the levels of USP7 protein were reduced by approximately 90% in the sh-USP7 group in comparison to the sh-control group. As anticipated, the knockdown of USP7 was observed to suppress the protein expressions of VCAM-1, ICAM-1 and E-selectin in both HUVECs (Figs. 2G and S1C) and HLMECs (Figs. 2H and S1D). Moreover, the silencing of USP7 also resulted in a reduction in the mRNA levels of VCAM-1, ICAM-1, IL-6 and IL-1β in activated endothelial cells (Fig. 2I). Similarly, the knockdown of USP7 was observed to markedly reduce the adhesion of THP-1 cells to HUVECs and HLMECs (Fig. 2J–L), respectively. These collective results indicated that USP7 exerts a positive modulatory effect on TNFα-induced EC activation.

These collective results indicated that USP7 exerts a positive modulatory effect on TNFα-induced EC activation.

### Inhibition of USP7 suppressed TNFα-induced endothelial activation

**Fig. 3:**
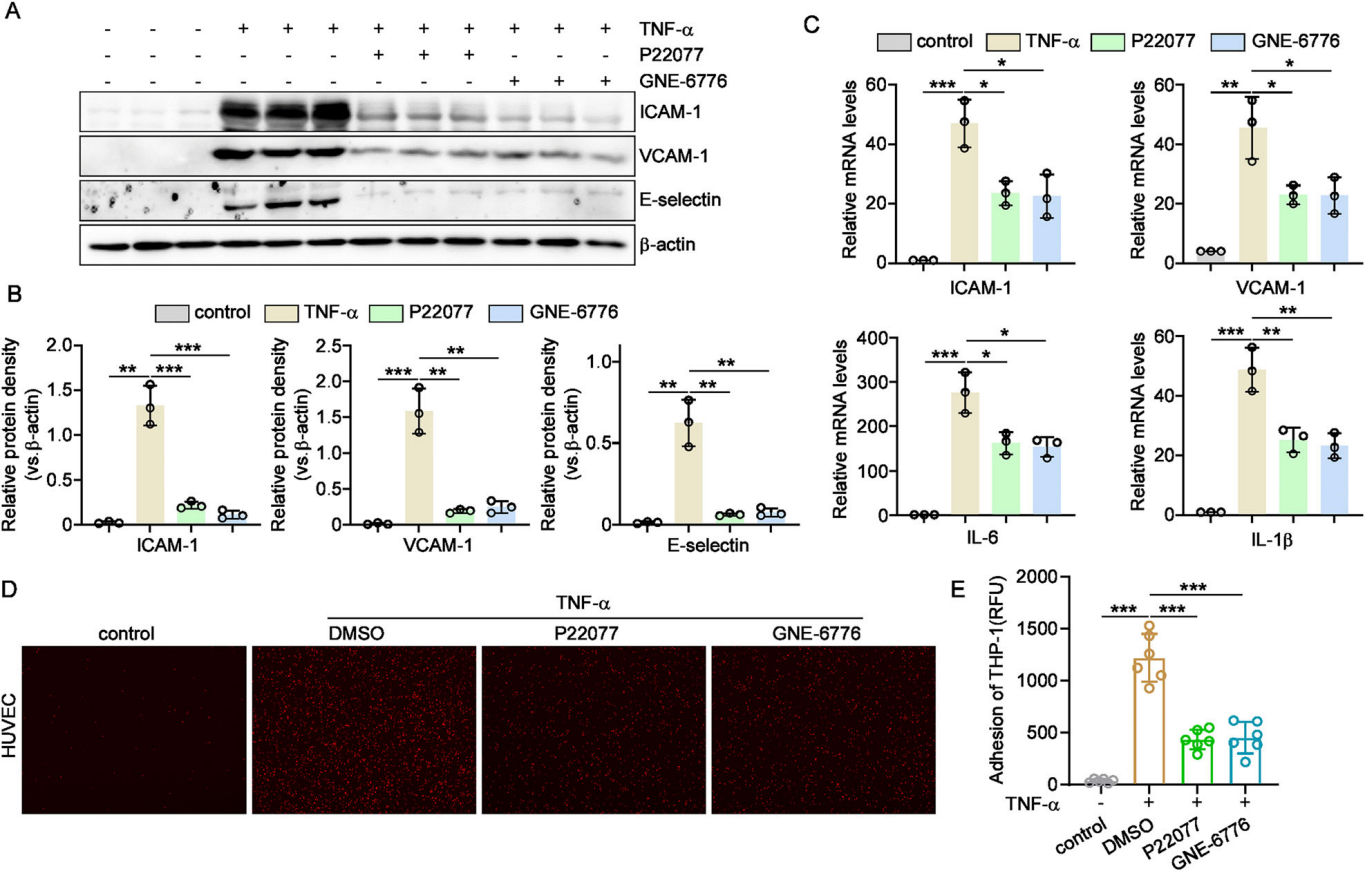
The effect of USP7 inhibitors on the activation of ECs. **A** After treatment of USP7 inhibitor P22077 (10 μM) and GNE-6776 (15 μM) for 24 h, HUVECs were then incubated with 10 ng/mL TNFα for another 8 h and cell lysates were collected to detect the protein levels of adhesive molecules, including ICAM-1, VCAM-1 and E-selectin; **B** Relative fold changes of proteins were determined by Gel-Pro Analyzer software, normalized to β-actin; **C** After 4 h of TNFα treatment, the mRNA levels of adhesive molecules and cytokines were determined by qPCR in USP7 inhibitor treated HUVECs as indicated; **D** Upon incubation with TNFα for 8 h, USP7 inhibitor treated HUVECs were co-cultured with PKH-26 labeled THP-1 cells for 1 h and observed under fluorescent microscope; **E** Attached cells were counted from six random pictures. All data were expressed as mean ± SD. *P < 0.05, ** *P* < 0.01, ***P < 0.001.

As knockdown of USP7 was found to be significantly inhibitory to the activation of EC cells, the effect of USP7 inhibitors on TNFα-induced endothelial activation was investigated. As shown in Fig. 3A, B, administration of both USP7 inhibitor P22077 and GNE-6776 suppressed the protein levels of key adhesive molecules in HUVECs as indicated. Furthermore, the mRNA levels of ICAM-1, ICAM-1, IL-6 and IL-1β decreased after treatment of P22077 and GNE-6776 (Fig. 3C). As expected, the adhesion of THP-1 cells to HUVECs was markedly reduced by the inhibition of USP7 with P22077 and GNE-6776 (Fig. 3D, E). In summary, pharmacological inhibition of USP7 significantly suppressed the expression of adhesive proteins and cytokines, as well as the monocyte adhesion.

### Inhibition of USP7 protects against LPS-induced ALI

**Fig. 4:**
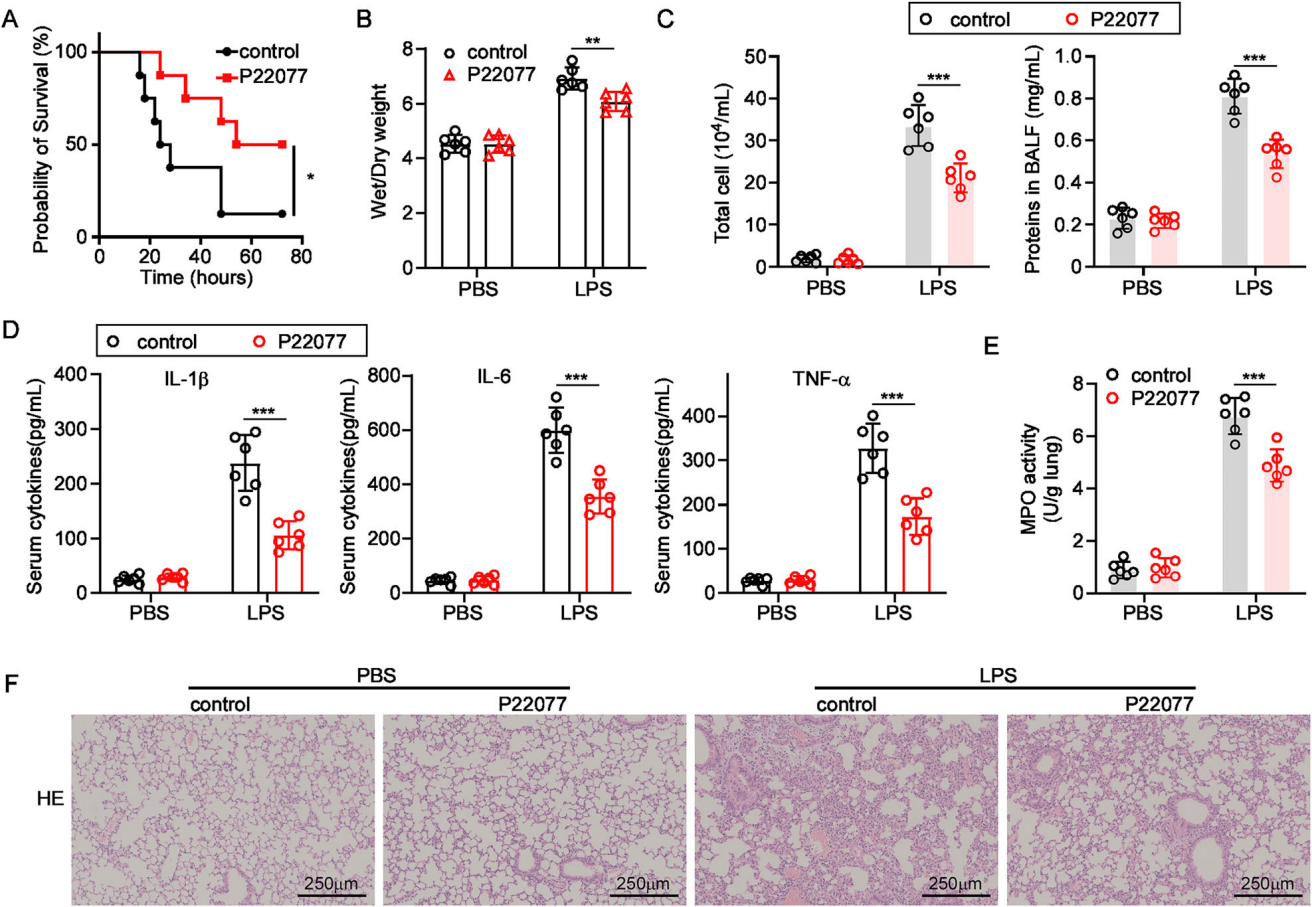
Effects of USP7 inhibitor on LPS induced ALI. **A** Effect of P22077 on the survival rate of mice challenged with 15 mg/kg body weight of LPS injection (i.p.); **B** The lung wet-to-dry ratio; **C** Total cell number and proteins from the BALF were detected in the PBS and P22077 treated groups; **D** The content of IL-1β, IL-6, and TNFα in the serum was detected by ELISA; **E** The MPO activities in lung tissues; **F** Representative images of HE staining of mice lungs in control and ALI groups. All data were expressed as mean ± SD. *P < 0.05, **P < 0.01, ***P < 0.001.

To investigate the effect of USP7 on mice ALI, an LPS-induced ALI model was performed using a selective inhibitor of USP7 in vivo. The inhibition of USP7 resulted in a prolonged survival rate and an extended period of survival following a high-concentration LPS challenge, in comparison to the control group (Fig. 4A). Following a 24-h challenge with a sublethal dose of LPS (5 mg/kg body weight), the extent of lung inflammation and injury was evaluated through the analysis of lung edema, histology, and leukocyte infiltration in bronchoalveolar lavage fluid (BAL). As illustrated in Fig. 4B, the administration of P22077 resulted in a notable reduction in pulmonary edema, as evidenced by a marked decrease in the wet/dry ratio. Furthermore, the mice that had been injected with P22077 displayed reduced levels of cells and protein in the BALF, which was a consequence of the LPS-induced ALI (Fig. 4C). The secretion of pro-inflammatory cytokines, including IL-1β, IL-6, and TNF-α, was also diminished when USP7 was inhibited (Fig. 4D). Furthermore, the treatment with the USP7 inhibitor resulted in a consistent inhibition of MPO activities in lung tissues (Fig. 4E). As illustrated in Fig. 4F, the administration of LPS led to the development of severe lung inflammation and injury in mice, whereas the treatment with P22077 significantly alleviated lung injury (Fig. 4F). These findings substantiate the notion that USP7 plays a pivotal role in ALI in vivo, thereby indicating its potential as a promising therapeutic target for ALI in clinical settings.

### USP7 interacts with PDK1 in endothelial cells

**Fig. 5:**
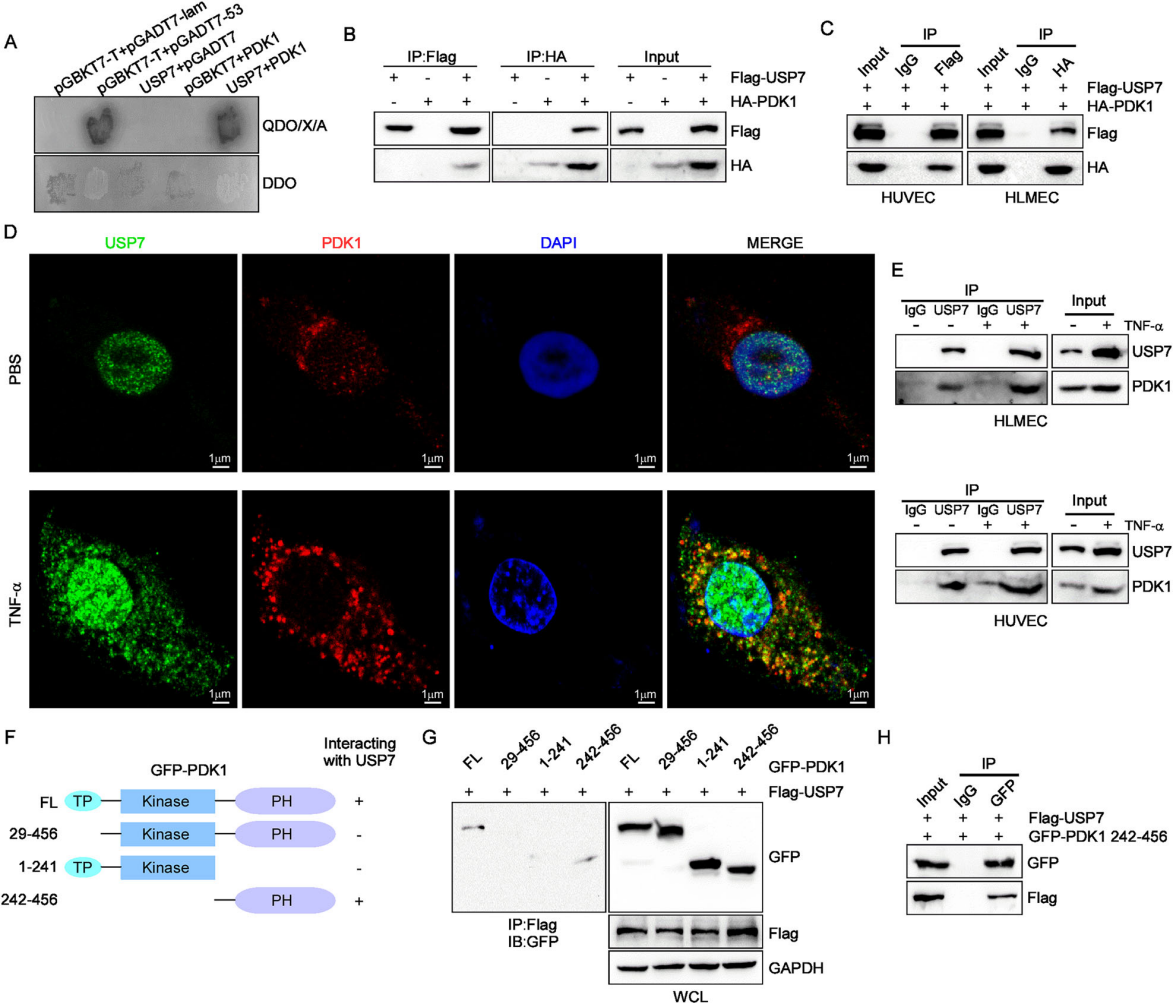
USP7 can bind to PDK1. **A** PDK1 was identified as a new binding protein of USP7 through the yeast-two-hybrid system; **B** Co-IP analysis of the interaction between Flag-USP7 and HA-PDK1 in HEK293T cells; **C** Co-IP analysis of the interaction between Flag-USP7 and HA-PDK1 in HUVECs and HLMECs; **D** Co-localization of endogenous USP7 and PDK1 in HUVECs; **E** Endogenous interaction of USP7 and PDK1 was enhanced after TNFα treatment both in HUVECs and HLMECs via co-IP assays using specific USP7 antibodies (normal IgG was used as negative control); **F** Schematic diagram of PDK1 deletion mutants generated in pCMV-EGFP vectors; **G** Interaction of USP7 with GFP-PDK1 and its deletion mutants was determined by co-IP assays with Flag antibodies; **H** Co-IP analysis of the interaction between Flag-USP7 and GFP-PDK1 (242–456) in HEK293T cells using GFP antibodies.

To gain insight into the molecular mechanism by which USP7 regulates ALI, we employed a yeast-two-hybrid system to screen for interacting proteins of USP7. Among the candidate proteins, the 3-phosphoinositide dependent protein kinase-1 (PDK1) was selected for further investigation, as it has been previously demonstrated to be involved in LPS-induced ALI through NF-κB signaling pathway activation [14]. Yeast cells expressing both USP7 and PDK1 were observed to grow on QDO plates in a manner similar to that of positive controls, indicating a strong interaction between the two proteins (Fig. 5A). Subsequently, the interaction between USP7 and PDK1 was validated through a co-immunoprecipitation (co-IP) assay utilizing cell lysates from transfected HEK293T cells, as illustrated in Fig. 5B. In both endothelial cell lines tested (HUVECs and HLMECs), ectopic USP7 was observed to interact with PDK1 in a manner consistent with the results in HEK293T cells (Fig. 5C). It is noteworthy that treatment with TNF-α resulted in a notable elevation in USP7 expression. Furthermore, the co-localization of endogenous PDK1 and USP7 was also enhanced in the cytoplasm (Fig. 5D). Similarly, the endogenous binding of PDK1 and USP7 was observed to increase upon TNFα incubation (Fig. 5E).

Based on the structural characteristics of PDK1, a series of plasmids with various domain mutants was generated, as illustrated in Fig. 3F. The resulting plasmids were then used to perform co-immunoprecipitation assays to identify the domain responsible for the interaction with USP7. In addition to the full-length PDK1, the C-terminus of PDK1 with the PH domain was found to be the only domain capable of binding to USP7 (Fig. 5G). The interaction between Flag-USP7 and GFP-PDK1 (242-456) was further confirmed via Co-IP assays using GFP antibodies (Fig. 5H). These findings collectively demonstrate that USP7 can interact with PDK1.

### USP7 deubiquitinates and stabilizes PDK1 in endothelial cells

**Fig. 6:**
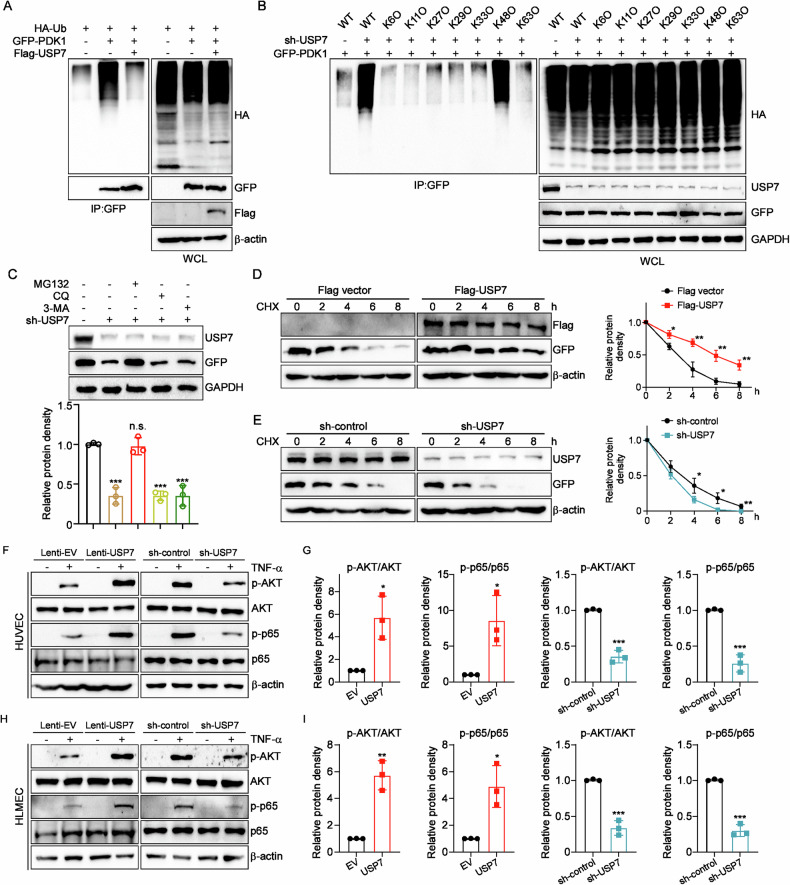
USP7 stabilized PDK1 in a UPS-dependent manner. **A** HEK293T cells were co-transfected with HA-Ub, GFP-PDK1/GFP and Flag-USP7/Flag for 24 h; **B** HEK293T cells were co-transfected with GFP-PDK1, sh-USP7 or sh-control plus different HA-Ub mutants (WT, K6O, K11O, K27O, K29O, K33O, K48O, K63O) for 24 h. Cell lysates were harvested and immunoprecipitated with anti-GFP antibody, and were also detected using the indicated antibodies by western blot (**A**, **B**); **C** HEK293T cells were co-transfected with GFP-PDK1 and sh-USP7 for 24 h, and then were treated with proteasome inhibitor MG132, lysosome inhibitor chloroquine (CQ) and autophagy inhibitor 3-MA for another 8 h. Protein level of PDK1 was assessed by western blot with GAPDH as the loading control; **D** Overexpression of USP7 prolonged the degradation of PDK1 after CHX treatment; **E** Knockdown of USP7 accelerated the degradation of PDK1 after CHX treatment; **F** Representative western blot images of indicated proteins in HUVECs overexpressing or silencing USP7; **G** The relative protein density was determined using Gel-Pro Analyzer software, normalized to total protein levels; **H** Representative western blot images of indicated proteins in HLMECs overexpressing or silencing USP7; **I** The relative protein density was determined using Gel-Pro Analyzer software, normalized to total protein levels. Relative fold changes were determined using Gel-Pro Analyzer software, normalized to GAPDH or β-actin. All data were expressed as mean ± SD. *P < 0.05, **P < 0.01, ***P < 0.001.

Given that USP7 has been demonstrated to function as a deubiquitinase in the regulation of numerous cellular processes, we proceeded to investigate its role in the ubiquitination levels of PDK1. As illustrated in Fig. 6A, the overexpression of USP7 was observed to markedly suppress the ubiquitination of PDK1. Subsequently, the lysine linkage pathway of ubiquitination of PDK1 was investigated through the use of seven distinct Ub mutants (K6O, K11O, K27O, K29O, K33O, K48O, K63O), as illustrated in Fig. 6B. The knockdown of USP7 was observed to only promote the ubiquitination of PDK1 in the presence of K48O ubiquitin, indicating that USP7 deubiquitinates PDK1 in a K48-linked manner. K48-linked ubiquitination invariably results in protein degradation via the ubiquitin-proteasome system (UPS). Subsequently, the impact of USP7 on PDK1 protein levels was examined using a range of inhibitors. The results of Fig. 6C demonstrated that the decline in PDK1 protein levels following RNAi of USP7 could be reversed by the proteasome inhibitor MG132, which was consistent with the linkage way of Ub. Furthermore, the overexpression of USP7 was observed to enhance the stability of PDK1 in a time-dependent manner, whereas its knockdown was found to reduce the protein half-life of PDK1 (Fig. 6D, E).

Given that PDK1 acts as a master kinase of the protein kinase AGC family, predominantly governing cell survival, proliferation and metabolic homeostasis, we proceeded to detect the phosphorylation levels of its typical target proteins, p65 and AKT. As anticipated, the ectopic overexpression of USP7 resulted in elevated protein levels of phospho-p65 and phospho-AKT in both HUVECs and HLMECs (Fig. 6F-I). Conversely, the downregulation of USP7 via shRNA yielded the opposite outcomes. In conclusion, USP7 stabilizes PDK1 in ECs via deubiquitylation and modulates the activation of PDK1-related signaling pathways, such as NF-κB and AKT.

### USP7 targets PDK1 to regulate TNFα-induced endothelial activation

**Fig. 7:**
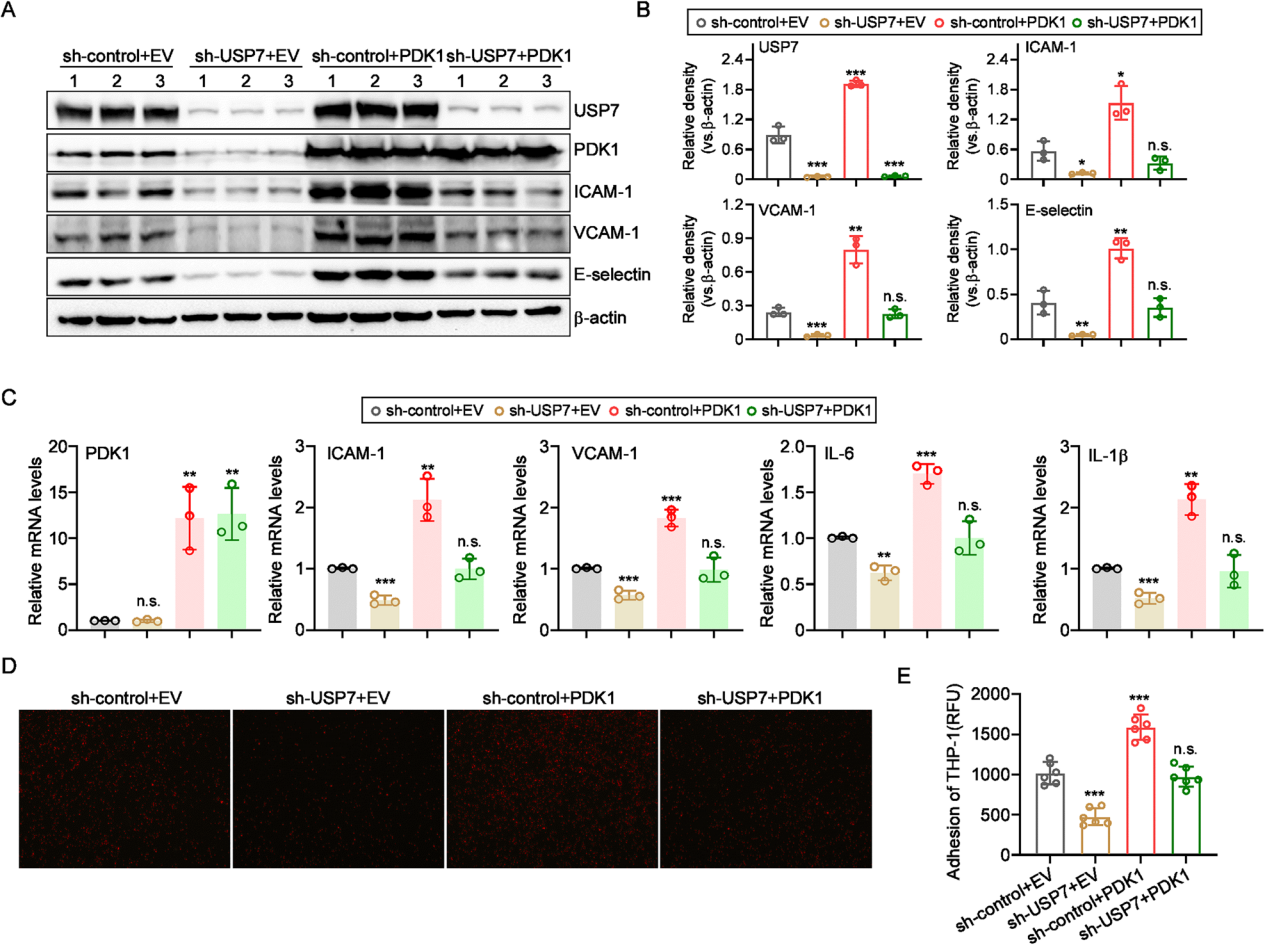
Knockdown of USP7 inhibited ECs activation dependent of PDK1. **A** Four groups of transfected HUVECs were treated with 10 ng/mL TNFα for 8 h, and then cell lysates were collected to detect the protein levels of adhesive molecules as indicated; **B** Relative fold changes of proteins were determined by Gel-Pro Analyzer software, normalized to β-actin; **C** After 4 h of TNFα treatment, the mRNA levels of adhesive molecules and cytokines were determined by qPCR in the above mentioned groups of HUVECs; **D** Upon incubation with TNFα for 8 h, HUVECs were co-cultured with PKH-26 labeled THP-1 cells for 1 h and observed under fluorescent microscope; **E** Attached cells were counted from six random pictures. All data were expressed as mean ± SD. N.s., *P* > 0.05, *P < 0.05, **P < 0.01, ***P < 0.001.

To investigate whether PDK1 is involved in the regulation of ECs activation by USP7, HUVECs were co-transfected with sh-USP7 and/or HA-PDK1 plasmids to knockdown the expression of USP7 and/or ectopic overexpression of PDK1. As shown in Fig. 7A, B, RNAi of USP7 resulted in a reduction in the protein levels of PDK1, ICAM-1, VCAM-1 and E-selectin, as anticipated. This effect could be reversed by the up-regulation of PDK1. Furthermore, the reduction in mRNA levels of these adhesive molecules and cytokines resulting from USP7 knockdown was also reversed when PDK1 was overexpressed (Fig. 7C). Additionally, the adhesive ability of HUVECs was enhanced with the supplementation of PDK1 (Fig. 7D, E). These findings collectively indicate that the endogenous knockdown of USP7 suppresses the activation of ECs via PDK1. RNAi of USP7 suppressed the protein levels of PDK1, ICAM-1, VCAM-1 and E-selectin as expected, which can be reversed by up-regulation of PDK1. Moreover, the decline of mRNA levels of those adhesive molecules and cytokines induced by USP7 knockdown was also improved when PDK1 was over-expressed (Fig. 7C). Consistently, the adhesive ability of HUVECs was enhanced with the supplement of PDK1 (Fig. 7D, E). The collective data indicated that endogenous knockdown of USP7 suppressed the activation of ECs via PDK1.

## Discussion

The pulmonary endothelium constitutes a semi-permeable barrier that is indispensable for pulmonary gas exchange at the alveolar-capillary interface and for regulating the passage of fluid and solute between the blood and the interstitium [15]. A disruption of the lung endothelial barrier function represents the most significant pathophysiological change observed in ALI [15]. Furthermore, pulmonary endothelial inflammation results in leukocyte adhesion, platelet aggregation and coagulation activation, which in turn leads to cell death, microthrombosis and fibrin deposition, thus increasing vascular permeability [16]. While pulmonary endothelial cells are not a primary source of cytokine release, they play a pivotal role in regulating innate cellular and cytokine responses [17]. Activation of endothelial cells results in the expression of a variety of leukocyte adhesion molecules, including ICAM-1, VCAM-1, and selectins, which mediate interactions between endothelial cells and leukocytes, as well as between endothelial cells and platelets [18]. Additionally, activated endothelial cells secrete proinflammatory cytokines, such as TNF and IL-1β, which contribute to endothelial injury and barrier disruption [17]. Our findings indicate that the deubiquitinating enzyme USP7 plays a regulatory role in endothelial cell activation. Furthermore, we have demonstrated that targeting USP7 effectively inhibits acute lung injury, suggesting that it may represent a promising drug target.

In recent years, USP7 has emerged as a promising epidemiological target. P22077, a specific USP7 inhibitor identified through activity-based chemical proteomics, has demonstrated anti-tumor and anti-inflammatory effects [19, 20]. P22077 has been demonstrated to inhibit the NF-κB and MAPKs pathways, exerting anti-inflammatory effects in vitro and in vivo. These effects are achieved by promoting K48-linked ubiquitination and TRAF6 degradation [21]. Palazon Riquelme et al. [22] found that in macrophages, P22077 was observed to prevent the formation and activation of inflammatory vesicles through the inhibition of USP7 and USP47, as well as the reduction of ASC oligomerization and speckling. Additionally, Liu et al. [23] demonstrated that a USP7 inhibitor could reduce the inflammatory response in osteoarthritis through the inhibition of the NLRP3 inflammatory pathway. Another study demonstrated that P22077 may inhibit the formation of NLRP3 inflammatory vesicle complexes and affect the activation of the inflammatory vesicle pathway in COPD rats, thereby reducing the release of downstream inflammatory mediators and ultimately controlling the progression of COPD [20]. Furthermore, our recent research indicated that P22077 can target the inhibition of the TGF-β signaling pathway to attenuate the development of idiopathic pulmonary fibrosis [12]. In the present study, P22077 was observed to reduce endothelial cell activation and inflammatory responses, and effectively block endothelial cell adhesion to monocytes, thereby delaying the mortality of LPS-induced acute lung injury in mice.

We identified a novel binding protein of USP7, PDK1, by yeast two-hybridization. PDK1 is a regulatory protein kinase located in the mitochondrial membrane and a member of the A, G, C (AGC) protein kinase family [24]. PDK1 has been shown to be an upstream activator of a variety of downstream effectors and may be involved in a number of diseases [25]. PDK1 can phosphorylate the T-loop of Akt in the presence of PIP3 and acts as a unique kinase to catalyze T308 phosphorylation of Akt [26]. Studies have shown that PDK1 activity is closely related to the onset and progression of the inflammatory response, and inhibition of PDK1 suppresses the systemic inflammatory response [27]. Keke Yang et al. [28] found that PDK1 interference not only reduced lung injury scores and attenuated lung histopathological damage, but also increased minute ventilation, airway resistance and lung volumes. However, direct inhibition of PDK1 may cause potential side effects due to its wide range of physiological effects [29]. Therefore, elucidating the regulatory mechanisms of PDK1 may help to find more attractive therapeutic strategies. In this study, we found that USP7 mediates the deubiquitination of PDK1, stabilizing PDK1 while promoting AKT phosphorylation levels, which in turn activates the NF-κB pathway and ultimately exacerbates the abnormal activation of endothelial cells.

Ubiquitination represents a highly conserved post-translational modification across mammalian species, which involves the participation of three key enzymes: the ubiquitin-activating enzyme E1, the ubiquitin-conjugating enzyme E2, and the ubiquitin ligase E3 [30]. The process of ubiquitination entails the transfer of the C-terminal glycine of ubiquitin to the ε-NH₂ group of the lysine residue present in the substrate [30]. Ubiquitination can be classified into three principal categories: mono-ubiquitination, polyubiquitination and polyubiquitination, the latter of which results in protein hydrolysis and signal transduction [31]. Conversely, the process of ubiquitination can be reversed through the removal of the ubiquitin chain by deubiquitinating enzymes (DUBs), which results in the termination of the ubiquitination process and the maintenance of the expression of the substrate protein [8]. The interplay between ubiquitination and deubiquitination is pivotal in regulating a multitude of biological processes [8]. Qiwei Jiang and colleagues [32] have demonstrated that SPOP orchestrates the polyubiquitination-mediated degradation of PDK1. It is noteworthy that PDK1 is also subject to monoubiquitination, with USP4 serving as a mediator for the deubiquitination modification of this monoubiquitination [33]. Nevertheless, the deubiquitination modification of the polyubiquitinated chain of PDK1 has yet to be documented. The present study reveals that USP7 mediates the deubiquitination modification of the K48-linked polyubiquitination site of PDK1. However, it is important to note that there are some limitations to this study. Firstly, the role of USP7 in endothelial cell activation was not validated in endothelial cell-specific knockout USP7 mice. Secondly, the specific deubiquitination site of USP7-mediated PDK1 has not been identified.

In conclusion, the findings of this study indicate that inflammatory factors induce the expression of USP7 in endothelial cells, which then mediates the deubiquitination of PDK1. This subsequently activates the downstream AKT-NF-κB signaling pathway, thereby promoting endothelial cell activation. The targeted inhibition of USP7 has been demonstrated to effectively attenuate LPS-induced endothelial inflammation and acute lung injury.

## Materials and methods

### Reagents

The human recombinant TNF-α was obtained from PeproTech, while the LPS was purchased from Sigma. The following compounds were procured from Sigma-Aldrich: MG132, CHX, 3-MA and CQ. P22077 and GNE-6776 were procured from MCE. The following antibodies were used: USP7 (66514-1-Ig), PDK1 (18262-1-AP), GAPDH (60004-1-Ig), β-actin (60009-1-Ig), and GFP (66002-1-Ig). The antibodies 50430-2-AP, HA (66006-2-Ig, 51064-2-AP), and Flag (66008-4-Ig, 20542-1-AP) were procured from Proteintech. The antibodies specific for ICAM-1 (sc-1511), VCAM-1 (sc-13160), and E-selectin (sc-14011) were obtained from Santa Cruz Biotechnology. The phospho-p65 (3033), p65 (8242), phospho-AKT (4058), and AKT (9272) antibodies were procured from Cell Signaling Technology. Protein A/G beads were obtained from Beyotime Biotechnology, while qPCR primers were obtained from TSINGKE Biological Technologies.

### Cell culture, transfection and lentivirus infection

The human umbilical vein endothelial cells (HUVEC) and human lung microvascular endothelial cells (HLMEC) were procured from Lonza Walkersville Inc. and cultured in ECM medium in accordance with the manufacturer’s instructions (Sciencell). The human acute monocytic leukemia cell line THP-1 was procured from Procell and maintained in RPMI 1640 medium containing 10% FBS and 1% penicillin/streptomycin (ExCell Bio). The HEK293T cells were procured from CCTCC and maintained in DMEM supplemented with 10% FBS and 1% penicillin/streptomycin. All cells were cultured at 37 °C with 5% CO₂ in a humidified incubator. The HEK293T cells were transfected with the indicated plasmids using the standard calcium phosphate technique. The transfection of HUVECs with the listed plasmids was conducted via PEI (Polyethylenimine Linear MW 40000, YEASEN, China), in accordance with the instructions provided by the manufacturer. Recombinant lentiviruses expressing USP7 (pHAGE) and lentiviruses with USP7 knocked down (pLVX-puro-IRES-GFP) were constructed and packaged as previously described [34]. The sequences of the USP7 shRNA were as follows: CCCAAATTATTCCGCGGCAAA. HUVECs and HLMECs were infected in the growth medium at a multiplicity of infection (MOI) of 25–50 with the aforementioned lentiviruses and a negative control. Endothelial cells were treated with 10 ng/mL tumour necrosis factor alpha (TNFα) or 100 ng/mL lipopolysaccharide (LPS) for the indicated times, and mRNAs or proteins were extracted and detected.

### Animals

The male C57BL/6 mice, aged seven weeks and weighing 18–22 g, were sourced from Hunan SJA Laboratory Animal Co., China. Throughout the experimental period, the animals were permitted to drink and eat at their own discretion, were housed at a constant room temperature of 24 °C, and were maintained in a 12-hour circadian rhythm environment with alternating days and nights. All animal experimental procedures were approved by the Institutional Animal Care and Use Committee of The First Affiliated Hospital of Nanchang University (No. CDYFY-IACUC-202412QR002) and performed in accordance with the ARRIVE guideline. All methods were carried out in accordance with relevant guidelines and regulations.

### ALI mouse models

The mice were randomly divided into four groups of six mice each to investigate the effects of LPS-induced sepsis on acute lung injury. The experimental groups were as follows: control (PBS), P22077 (5 mg/kg), LPS (5 mg/kg) and LPS + P22077 (10 mg/kg). To obtain the survival curve for ALI, two groups of mice (8 mice per group) were administered a single dose of LPS at a lethal dose (15 mg/kg). Each group received a pretreatment with equal volumes of PBS or P22077 solution via intraperitoneal injection for one week. Lung tissues were collected 24 h after intraperitoneal injection of PBS or LPS and fixed in 4% PFA for histological analysis. Serum samples were stored at −80 °C for further analysis.

### Western Blot

Following a wash with PBS, the cells were harvested or lysed with either 2× SDS loading buffer or RIPA lysis buffer. Lung tissues were harvested and homogenized in RIPA lysis buffer with protease inhibitors (Beyotime Biotechnology, Shanghai, China). Following BCA quantitation (Glpbio, Shanghai, China), the whole cell lysates were analyzed via western blotting as previously described [35]. The protein density was analyzed via Gelpro 32 software and normalized to that of the endogenous housekeeping protein (GAPDH or β-actin).

### RT-qPCR

Total RNA was isolated from the tissue or cellular samples using TRIzol reagent, in accordance with the instructions provided by the manufacturer (Life Technologies, CA, USA). A total of 1 μg of RNA was reverse transcribed into the first strand of complementary DNA (cDNA) using a reverse transcription kit (TakaRa, Dalian, China). Reverse transcription-polymerase chain reaction (RT-PCR) was conducted on an ABI 7500 real-time PCR system (ABI, MA, USA) with a SYBR-Green PCR Master Mix Kit (Yeasen Scientific, Shanghai, China). β-actin was selected as an endogenous control, and the relative level of the indicator gene was calculated via the 2^−ΔΔCt^ method. The sequences of primers utilized are listed in Table 1.

**Table 1:** Primers used in this study.

Gene	NCBI Gene ID	Locus	Sequence (5′ to >3′)		Location
*USP7*	7874	NM_003470	Forward primer	CCGAGGACATGGAGATGGAAG	680–700
			Reverse primer	GCCAACTGGTGTCATCCTCC	798–817
*ICAM-1*	3383	NM_000201	Forward primer	CGTGAATGTGCTCTCCCCC	1453–1471
			Reverse primer	TCCCTTTTTGGGCCTGTTGT	1582–1601
*VCAM-1*	7412	NM_001078	Forward primer	TTGGATAATGTTTGCAGCTTCTCA	164–187
			Reverse primer	AGATGTGGTCCCCTCATTCGT	333–353
*IL-6*	3569	NM_000600	Forward primer	CATCCTCGACGGCATCTCAG	240–259
			Reverse primer	ACCAGGCAAGTCTCCTCATTG	381–401
*IL-1β*	3553	NM_000576	Forward primer	GCAGAAGTACCTGAGCTCGC	91–110
			Reverse primer	TCCTGGAAGGAGCACTTCATCT	179–200

### ELISA

The concentrations of IL-1β, IL-6, and TNFα in the serum were quantified using ELISA kits from Neobioscience Technology, following the instructions provided. Absorbance at 450 nm was measured using an iMark Microplate Absorbance Reader (Bio-rad), and sample concentration was determined by interpolation from absorbance curves generated by recombinant protein standards.

### MPO detection

The lung tissues were weighed and homogenized, and the resulting supernatant was used for the MPO activity assay (Nanjing Jiancheng Bioengineering Institute, Nanjing, China). The assay was conducted in accordance with the manufacturer’s instructions, and the absorbance was determined at 460 nm.

### Monocyte adhesion assay

The adhesion assay was conducted in accordance with the previously described methodology [36]. The negative control, USP7 overexpressing or knock-down endothelial cells, were seeded into 6-well plates and treated with 10 ng/mL TNFα for 8 h. During this period, THP-1 cells were labelled with fluorescein isothiocyanate using a PKH26 fluorescent staining kit (MKBio), following the manufacturer’s instructions. Subsequently, the labelled THP-1 cells were added to the activated HUVECs or HLMECs at a density of 5×10⁵ per well and incubated for one hour at 37 °C. Non-adherent cells were removed by gently washing with cold PBS. The images of adherent THP-1 cells and the number were determined under a Cytation 3 Cell Imaging Multi-Mode Reader (Biotek Instruments).

### Yeast-two-hybrid

The full-length cDNA of the human USP7 gene was subsequently subcloned and inserted into the pGBKT7 vector as bait, while the human PDK1 gene was subsequently inserted into the pGADT7 vector as prey. The indicated combinations of vectors were transformed into the Y2HGold yeast strain, including negative controls (pGBKT7-Lam+pGADT7-T) and positive controls (pGBKT7-53 + pGADT7-T), and plated onto DDO (SD/-Leu/-Trp) and QDO/X/A (SD/-Ade/-His/-Leu/-Trp/X-α-Gal) plates. The presence of colonies on the higher stringency agar plates QDO/X/A was indicative of an interaction.

### Co-immunoprecipitation

For the co-immunoprecipitation (co-IP) experiments, the endothelial cells (pretreated with or without TNFα or transfected HEK293T cells were lysed with radioimmunoprecipitation assay (RIPA) lysis buffer containing protease and phosphatase inhibitors. The IP was conducted in accordance with the previously described methodology [35]. The supernatant of the cell lysates was combined with 1 μg of antibody and incubated at 4 °C for 2–4 h. Subsequently, 100 μL of protein A/G beads (Beyotime Biotechnology, Shanghai, China) were added to each tube and incubated overnight at 4 °C with gentle rotation (10 r/min). Following a gentle washing step, the binding proteins were analysed by immunoblotting with antibodies specific for Flag, HA, USP7 or PDK1, according to the aforementioned method.

### Immunofluorescence

To detect the co-localization of USP7 and PDK1, HUVECs were pre-inoculated on glass slides one day prior to immunofluorescence (IF) detection. Once the cell density reached a range of 20% to 30%, the cells were fixed in 4% cold PFA for two hours and incubated with 0.5% Triton X-100 for 20 min. Subsequently, the cells were blocked with 5% bovine serum albumin, after which the USP7 primary antibody (1:500, Proteintech) and the PDK1 primary antibody (1:500, Proteintech) were incubated for 12 hours at 4 °C. On the subsequent day, Alexa Fluor 488/594 secondary antibodies were employed to bind to USP7/PDK1 for 2 hours at room temperature. Thereafter, the nuclei were stained with DAPI (Servicebio, Wuhan, China), and images of the slides were captured using a Zeiss LSM800 confocal microscope.

### Histological examination

For histological analysis, the tissues were fixed in 4% paraformaldehyde and subsequently paraffin-embedded. Tissue sections (4 μm) were stained with hematoxylin and eosin. The extent of tissue damage was evaluated by three pathologists in a blinded manner, as previously described. Immunostaining of USP7 was performed using the standard procedures described previously [37].

### Statistical analysis

All data analysis was performed using GraphPad Prism 9.0 software, and data were expressed as mean ± SD. Comparisons between two groups were performed by two-tailed unpaired Student’s *t*-test. *P*-value less than 0.05 was considered significant.

## Supplementary information

SUPPLEMENTAL MATERIAL

Full and uncropped western blots

## Data Availability

The source data and materials that support the findings of this study are available from the corresponding author upon reasonable request.
