# Supplementary material for: USP7 promotes endothelial activation to aggravate sepsis-induced acute lung injury through PDK1/AKT/NF-κB signaling pathway
Source: Cell Death Discov. 2025 Apr 17;11:183. doi: 10.1038/s41420-025-02481-1 (PMC12006344; doi:10.1038/s41420-025-02481-1)
Supplement: Supplementary file 1 — SUPPLEMENTAL MATERIAL [file 41420_2025_2481_MOESM1_ESM.pdf]

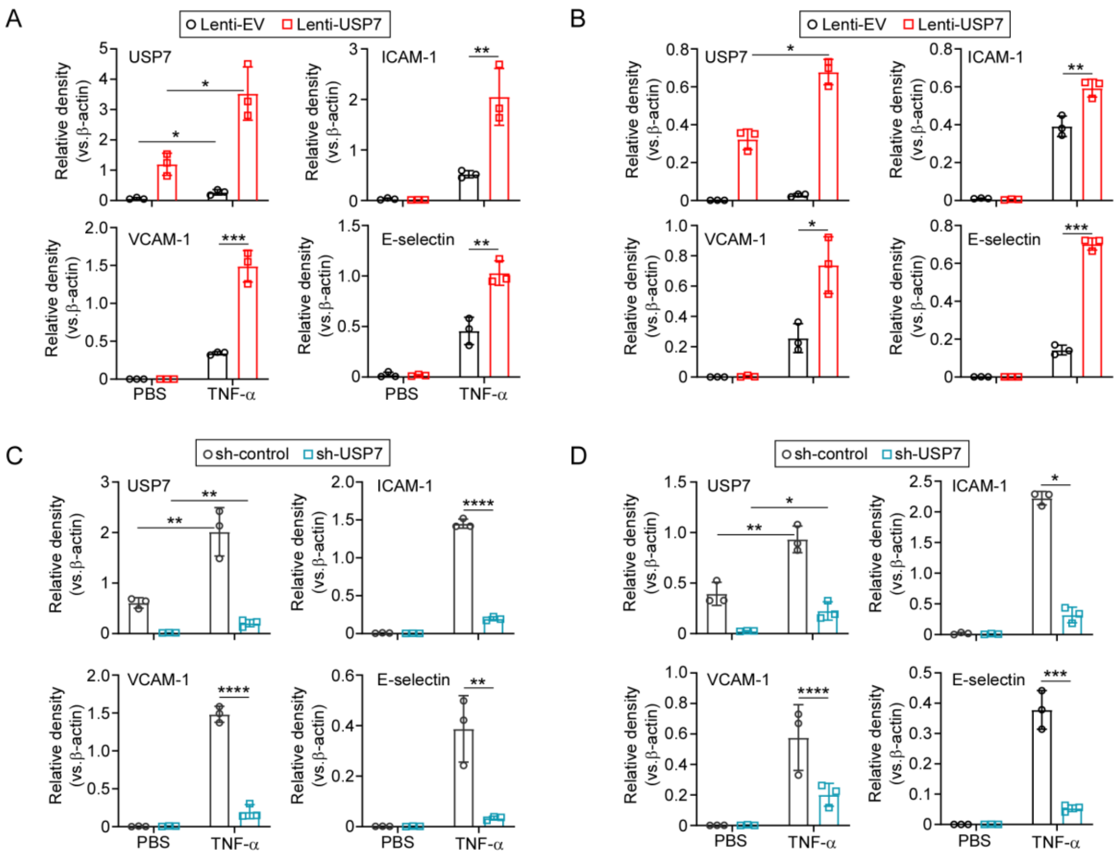

Figure S1. Quantitative analysis of the protein levels of adhesive molecules in HUVECs and HLMECs

Relative fold changes of proteins in Figure 2A (A), Figure 2B (B), Figure 2G (C) and Figure 2H (D) were determined by Gel-Pro Analyzer software, normalized to β-actin. All data were expressed as mean±SD. N.s., $P>0.05$, * $P<0.05$, ** $P<0.01$, *** $P<0.001$, **** $P<0.0001$.
