# Supplementary material for: USP7 promotes endothelial activation to aggravate sepsis-induced acute lung injury through PDK1/AKT/NF-κB signaling pathway
Source: Cell Death Discov. 2025 Apr 17;11:183. doi: 10.1038/s41420-025-02481-1 (PMC12006344; doi:10.1038/s41420-025-02481-1)

Full unedited pictures for WB results

Figure 1A

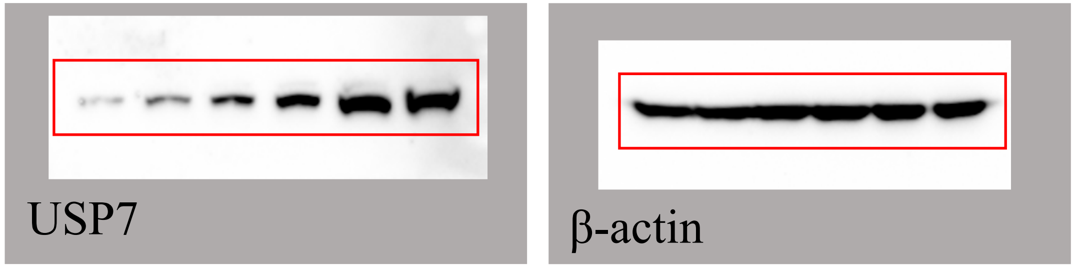

Figure 1C

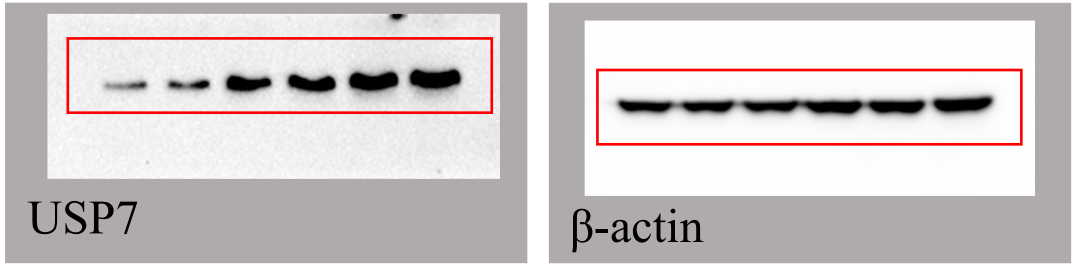

Figure 1B

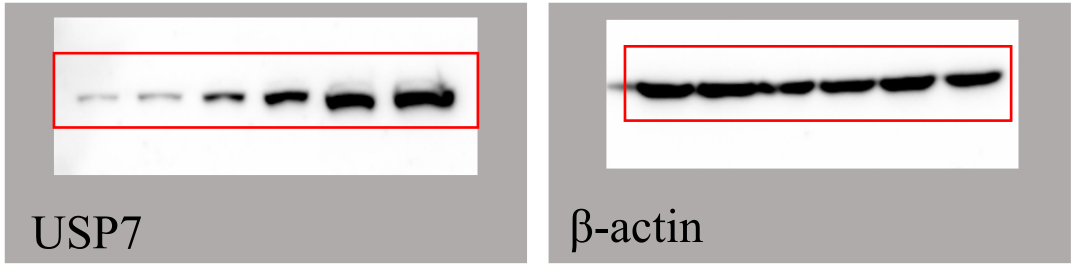

Figure 1D

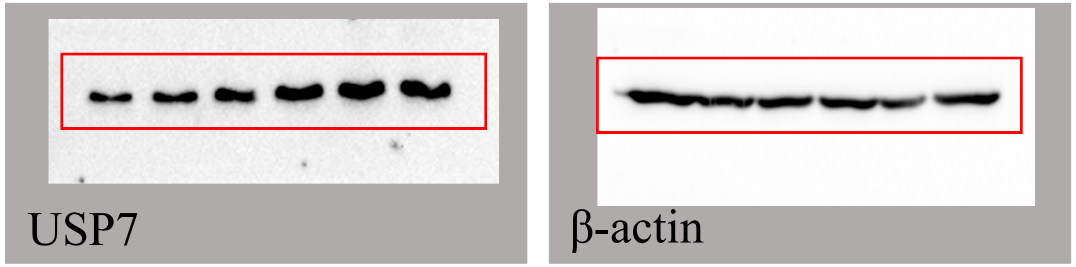

Figure 1F

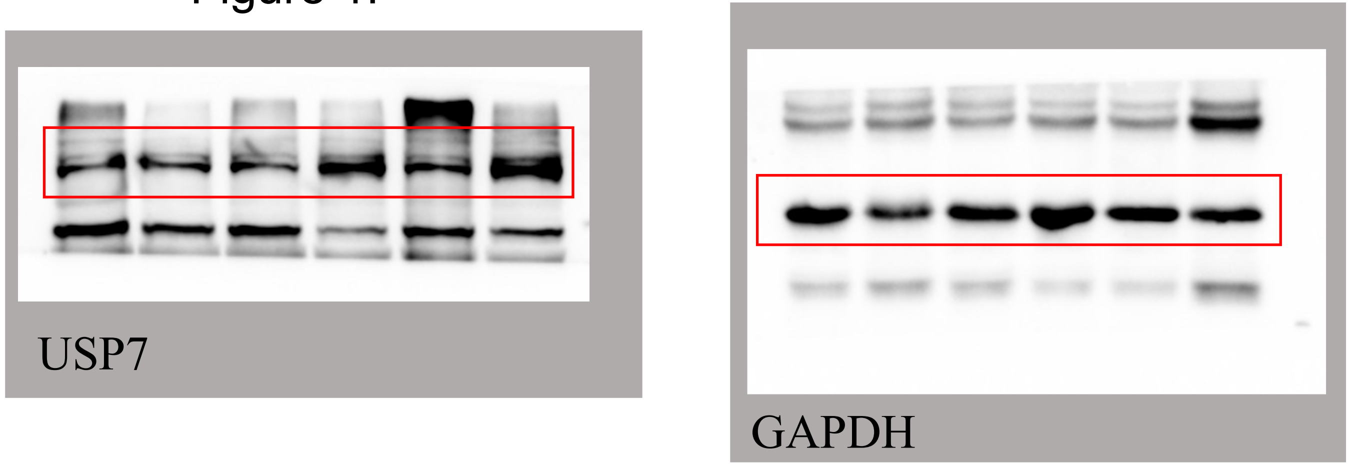

Figure 2A

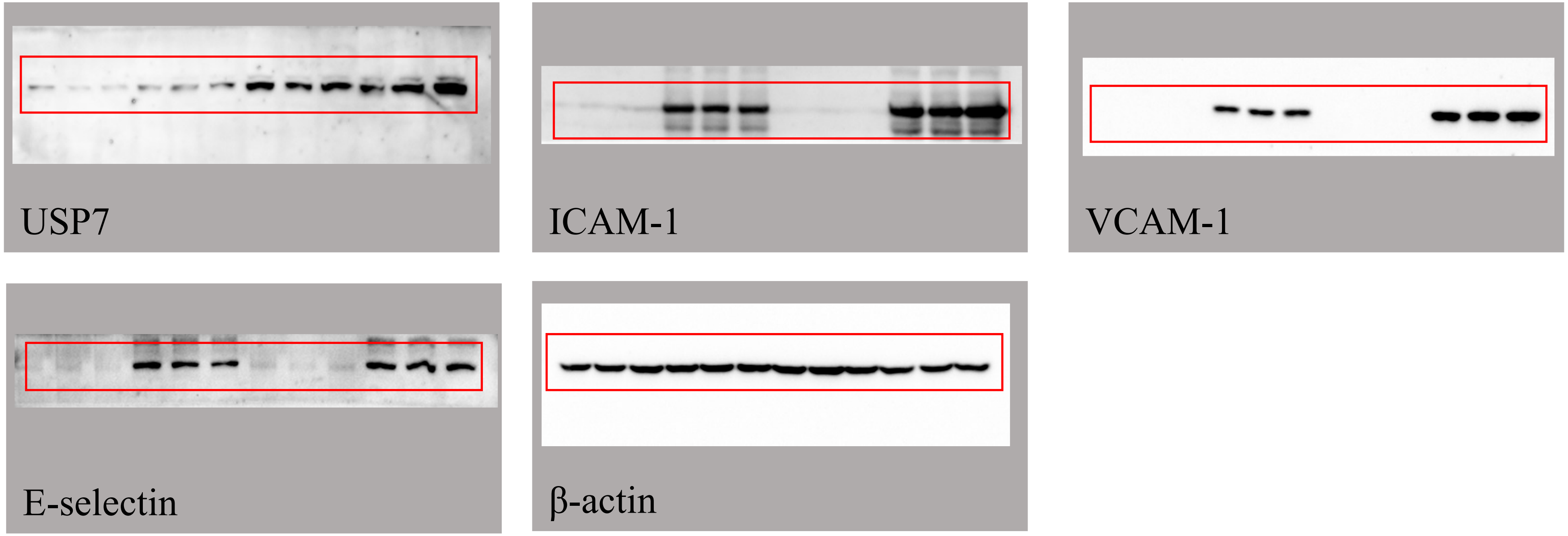

Figure 2B

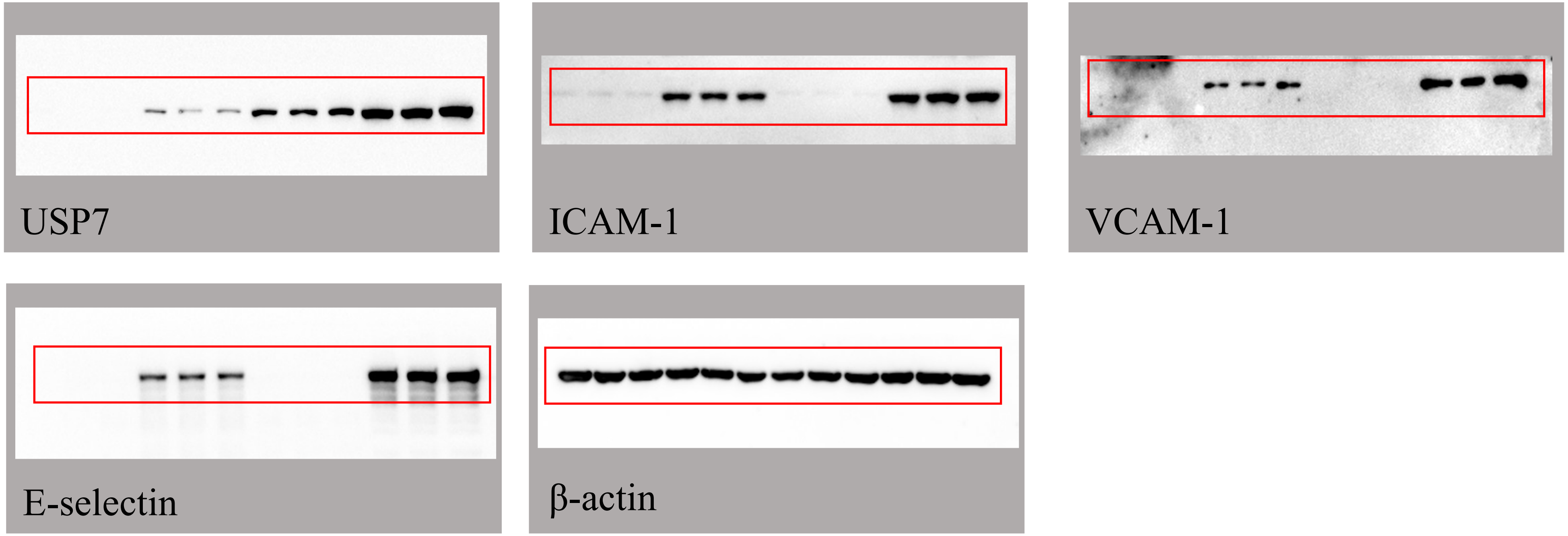

Figure 2G

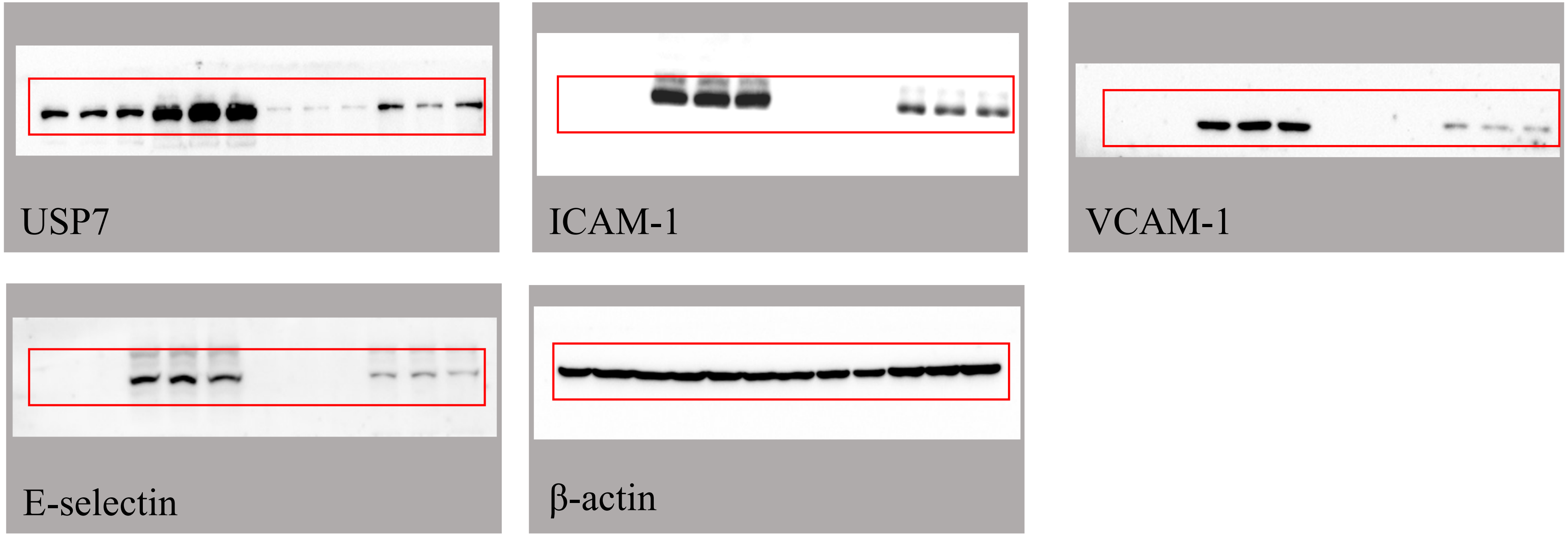

Figure 2H

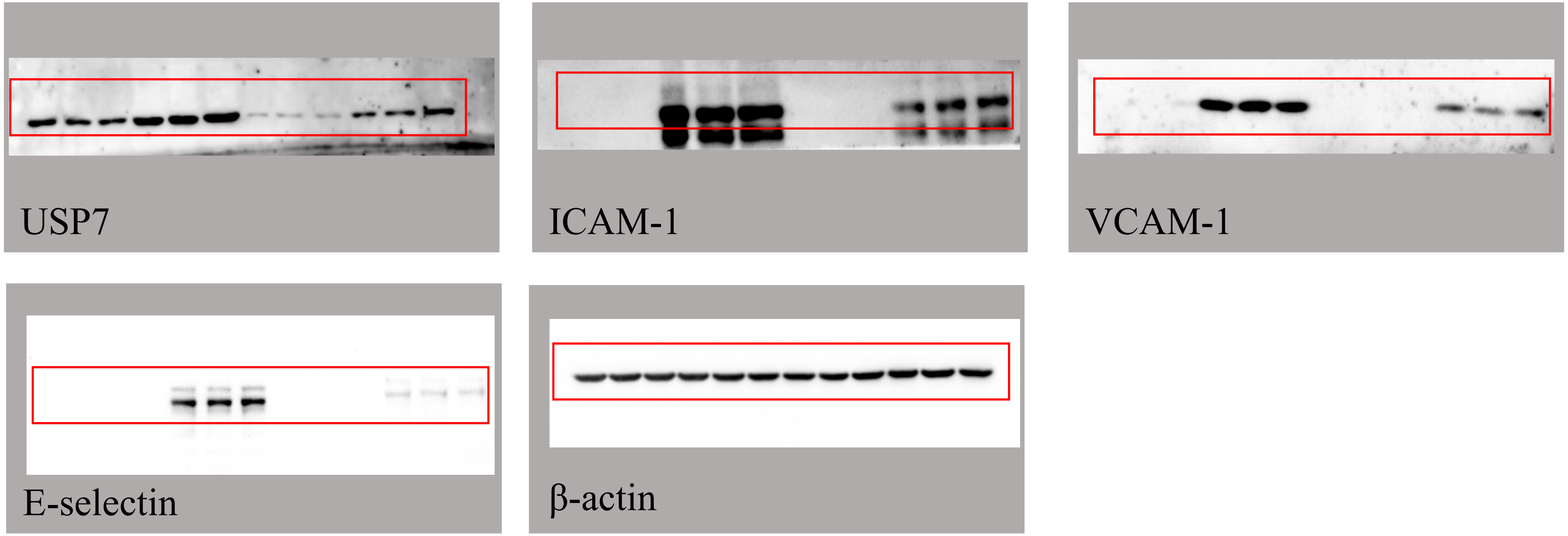

Figure 3A

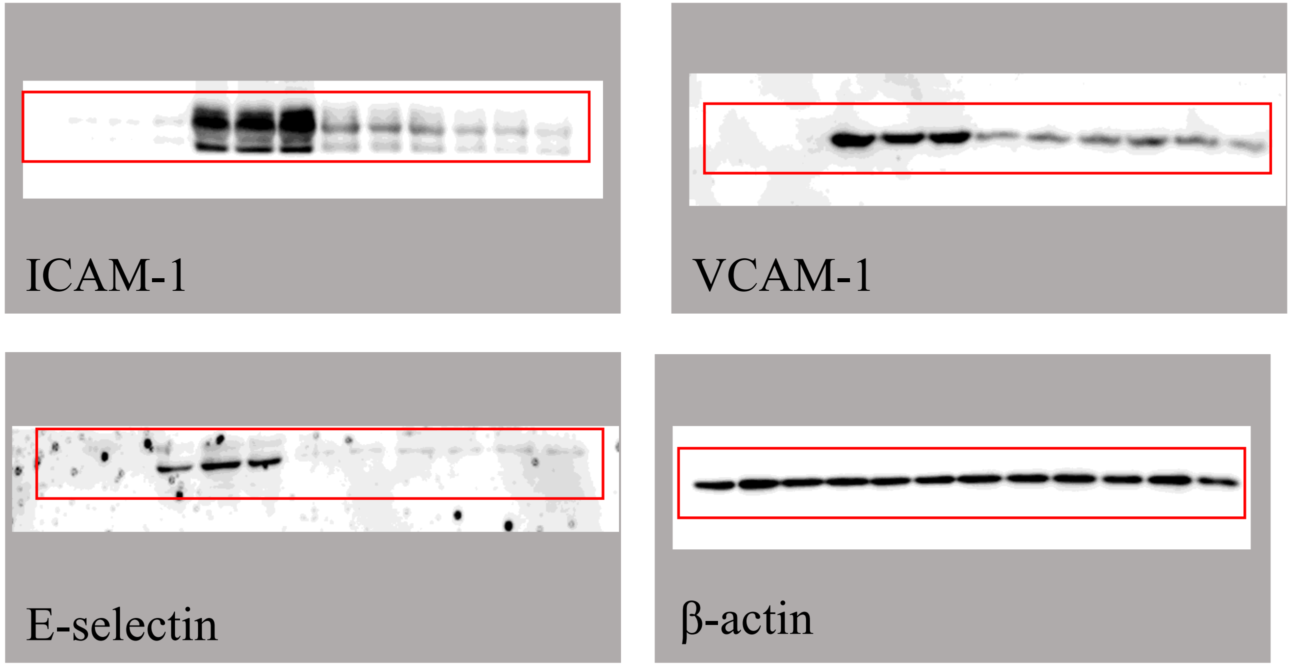

Figure 5B

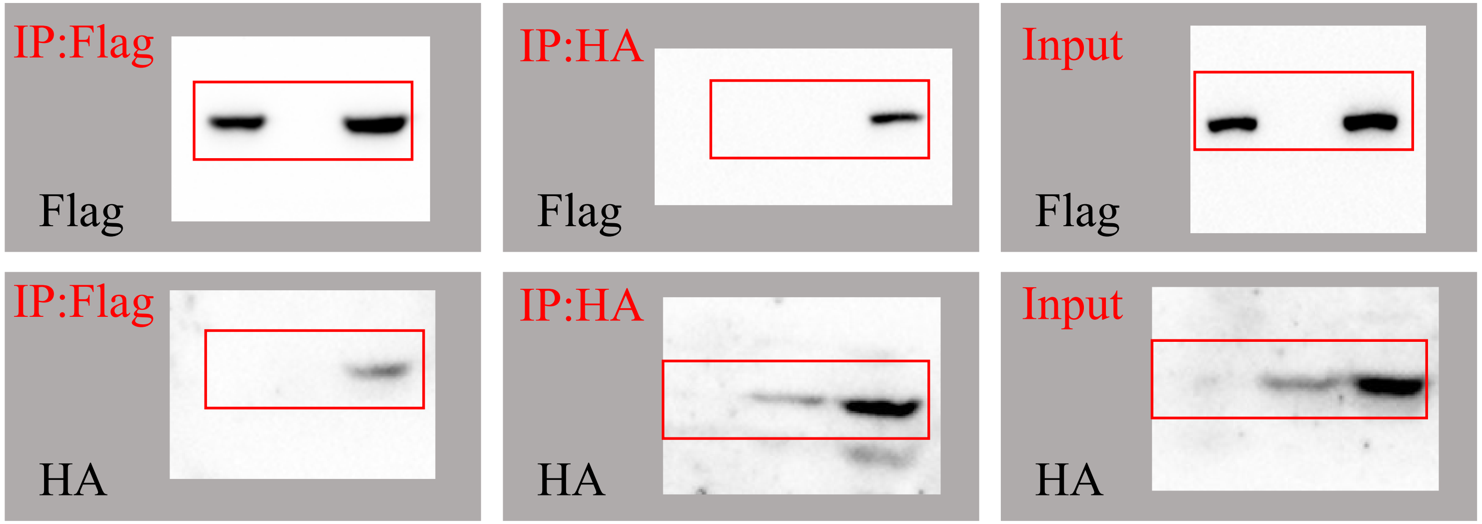

Figure 5C

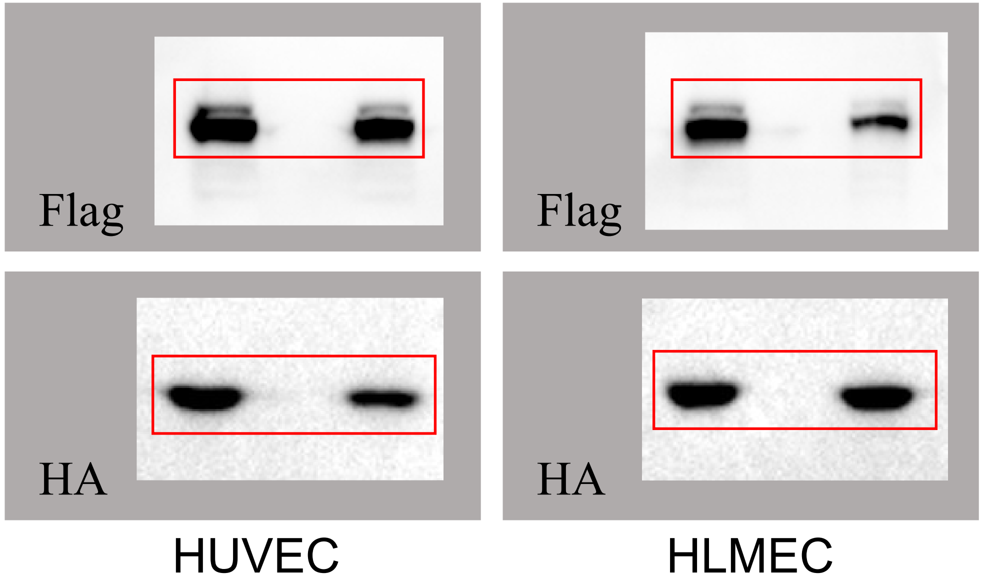

Figure 5E

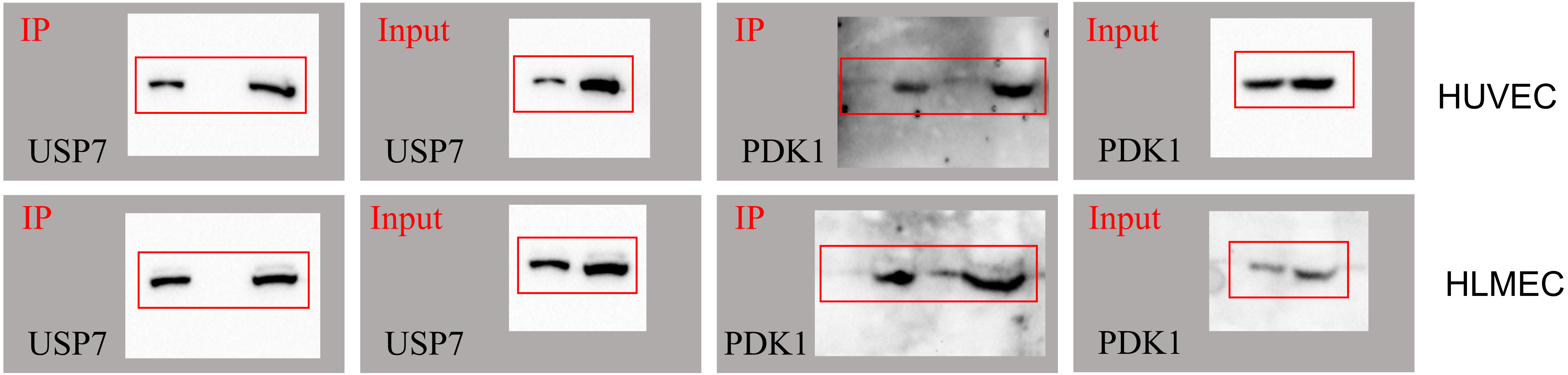

Figure 5G

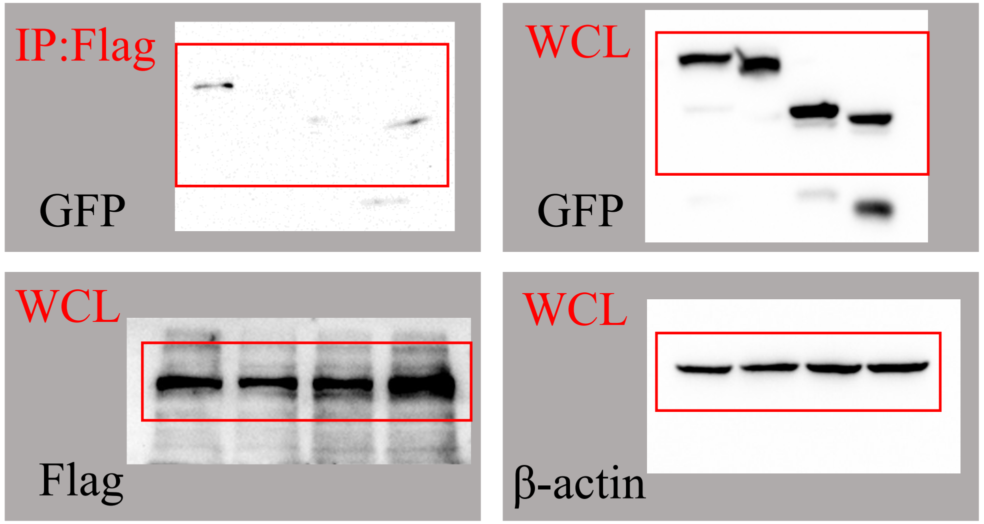

Figure 5H

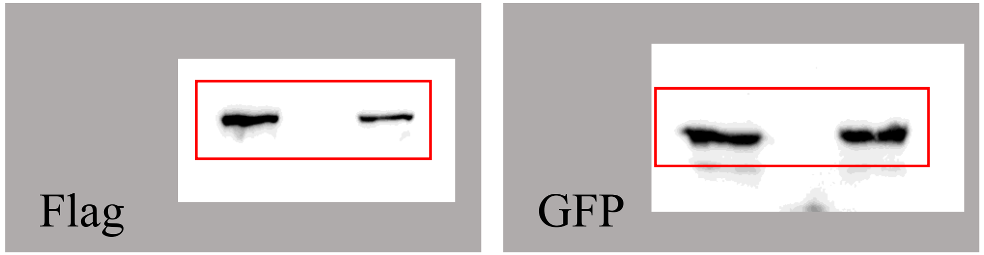

Figure 6A

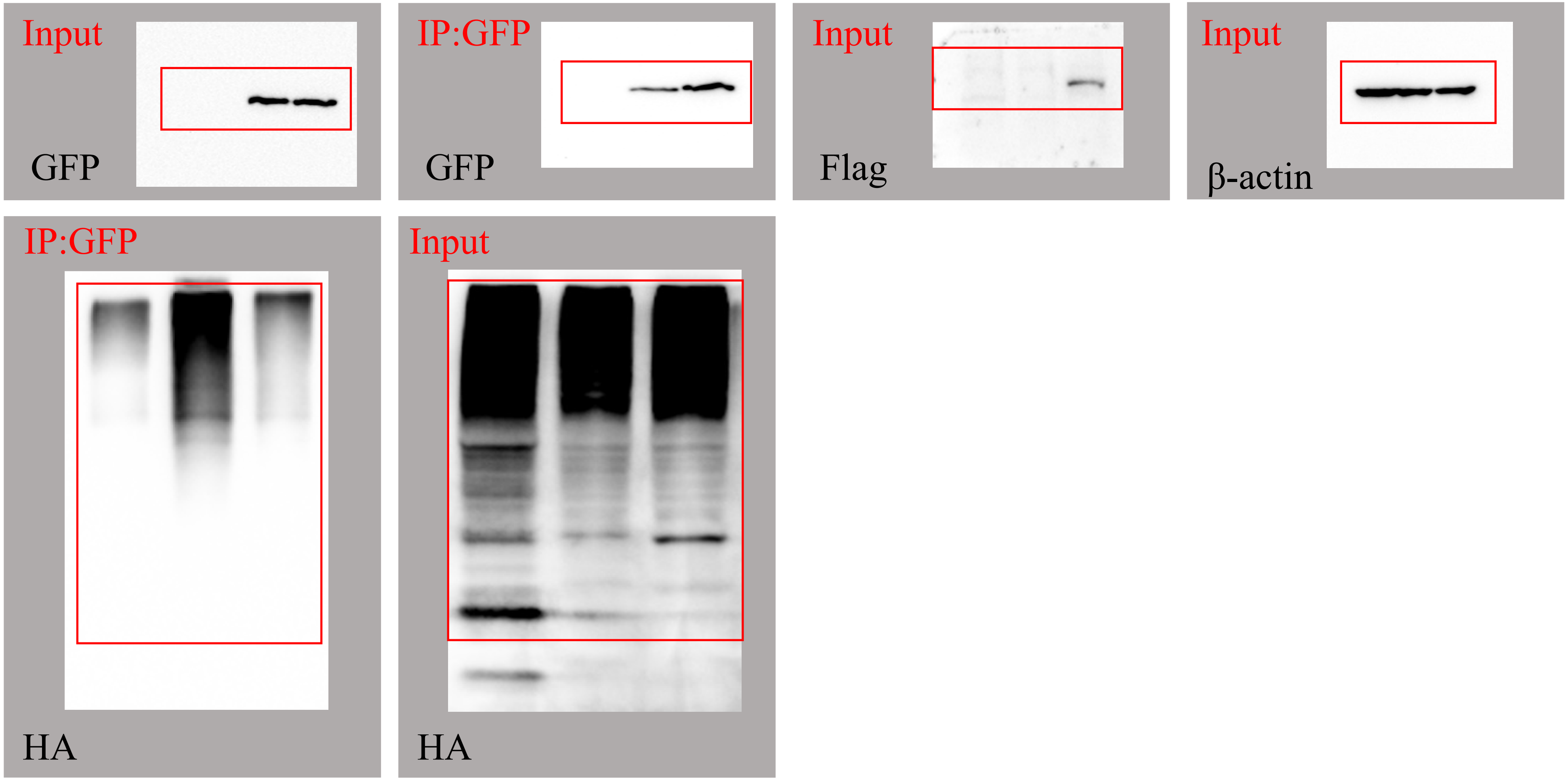

Input
GFP
IP:GFP
GFP
Input
Flag
Input
β-actin
IP:GFP
HA
Input
HA

Figure 6B

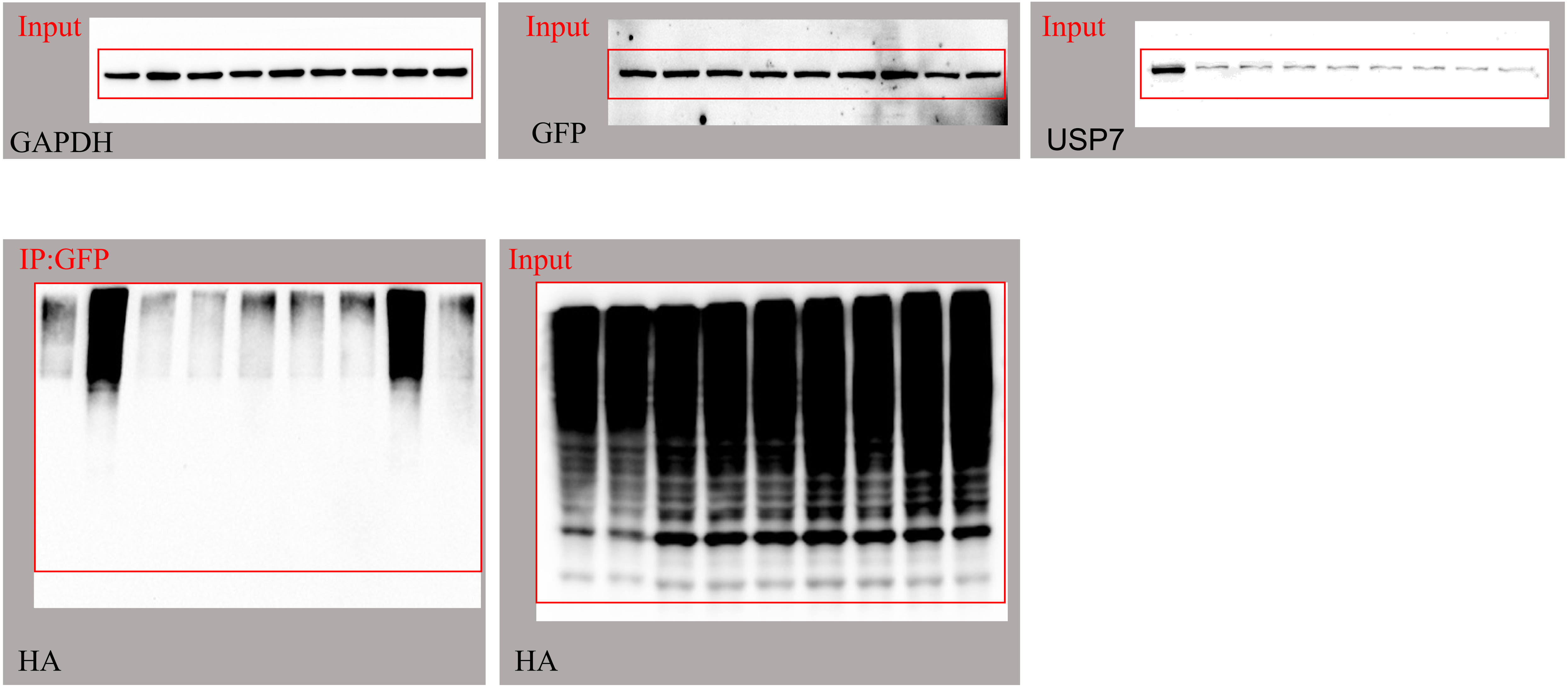

Figure 6C

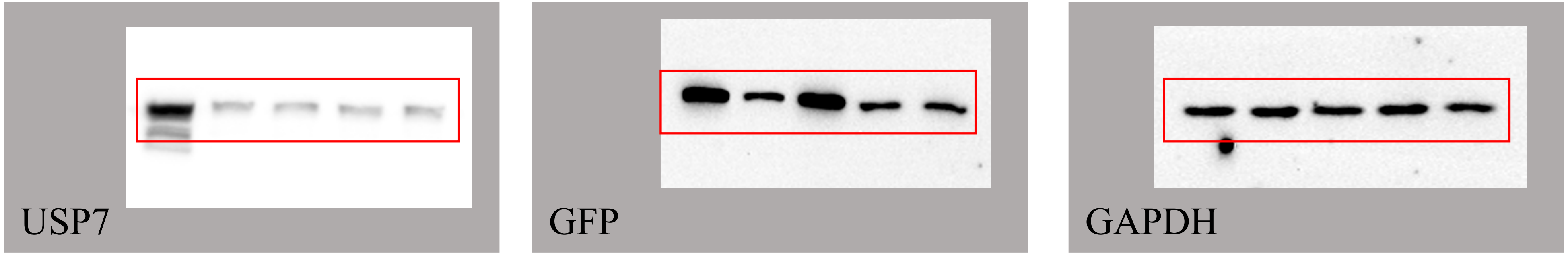

Figure 6D

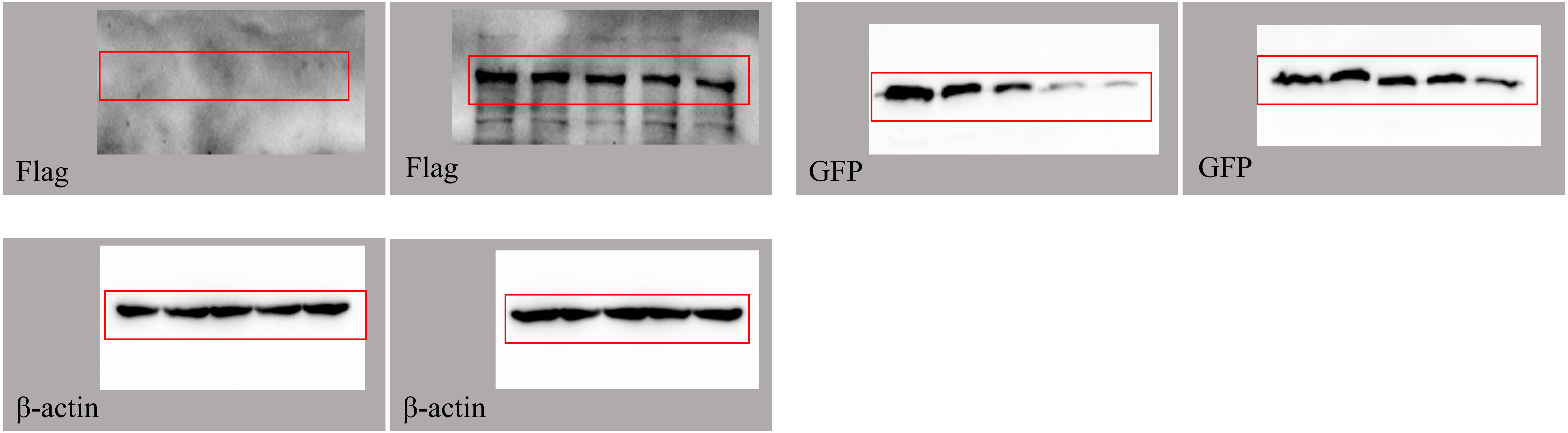

Figure 6D

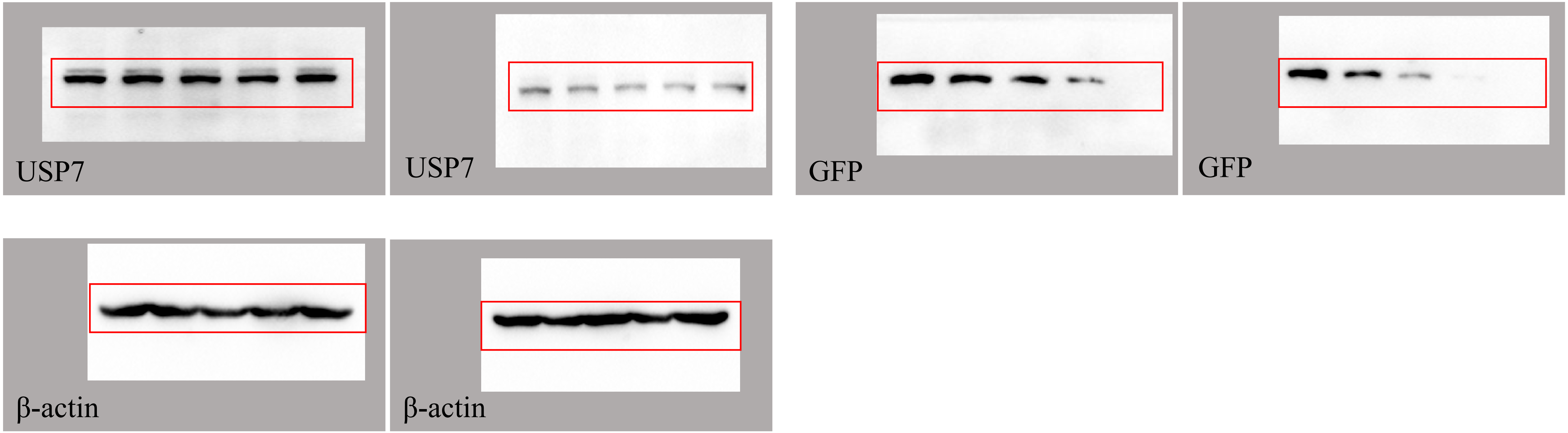

Figure 6F

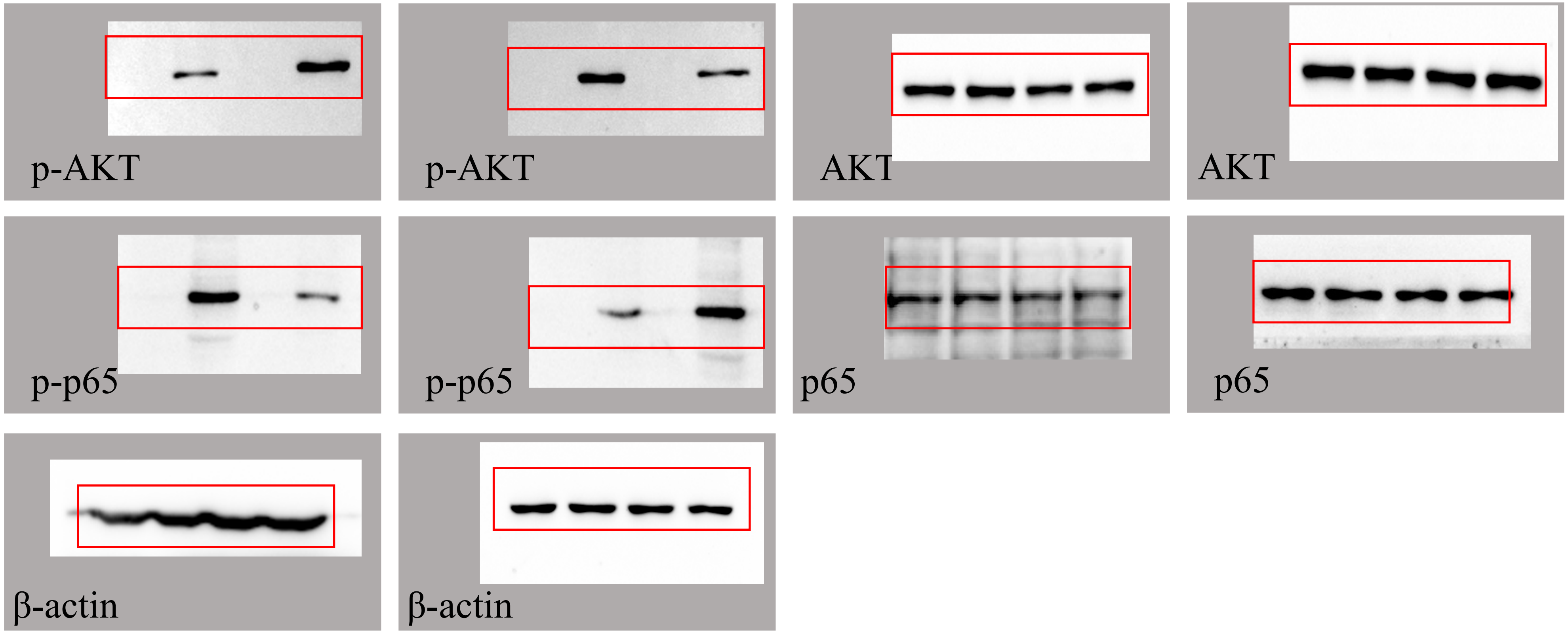

Figure 6H

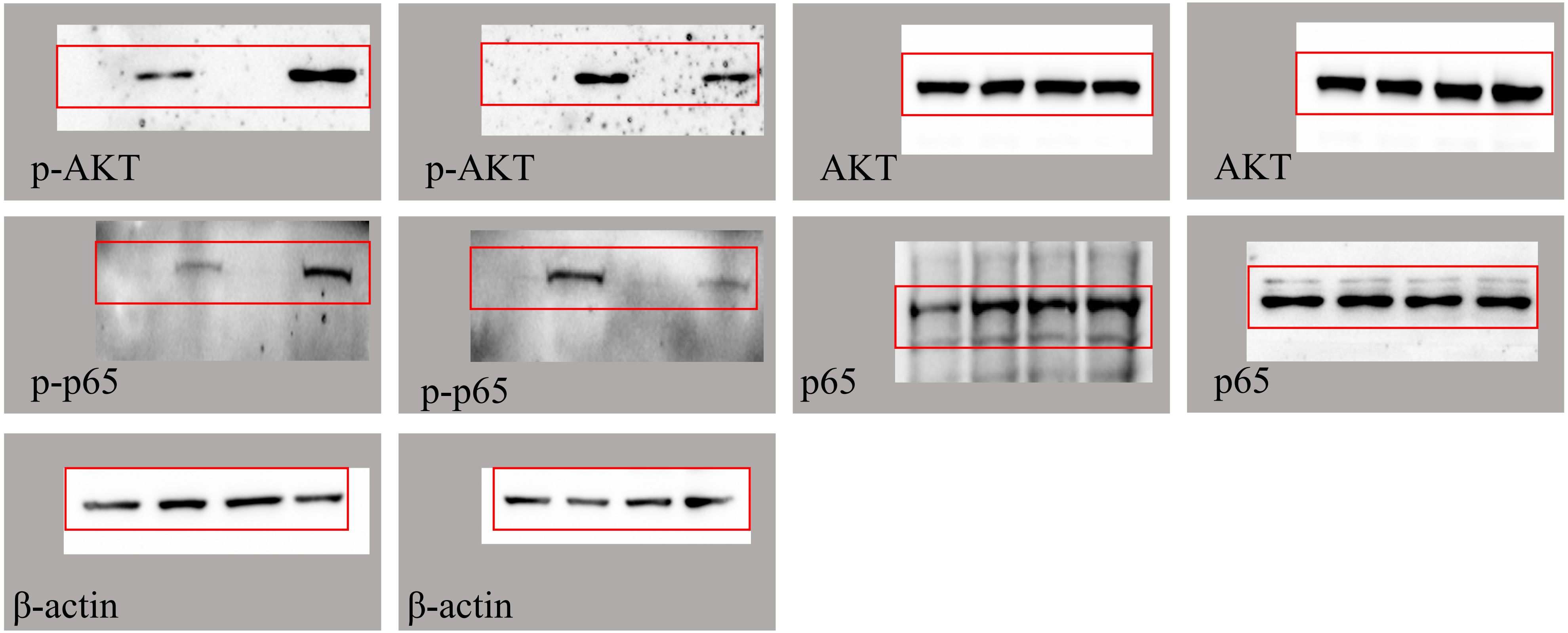

Figure 7A

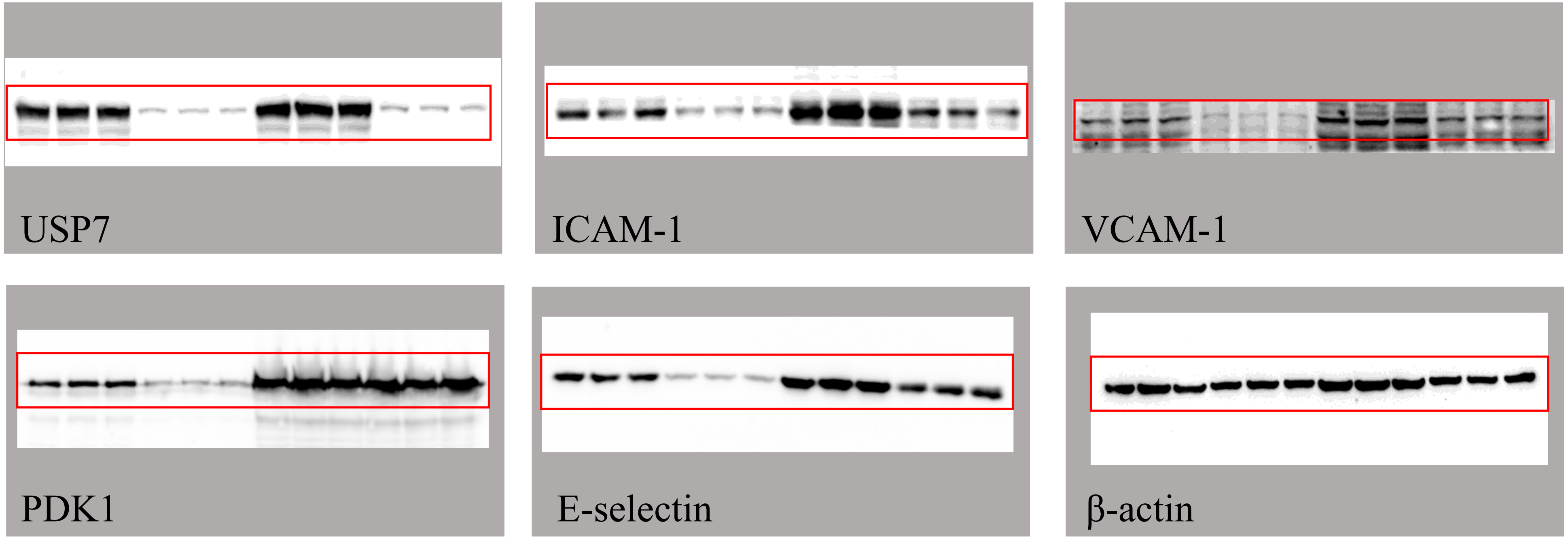

Supplement: Supplementary file 2 — Full and uncropped western blots [file 41420_2025_2481_MOESM2_ESM.pdf]
